# Risk Assessment of Neutropenia Based on Exposure Information Without Plasma Concentration Measurement in Pemetrexed–Platinum-Based Chemotherapy: A Modeling Approach Using Real-World Clinical Data

**DOI:** 10.3390/pharmaceutics18080946

**Published:** 2026-07-31

**Authors:** Kazunori Morita, Keiichi Shigetome, Haruka Narise, Tetsuya Kaneko, Naoto Soejima, Ryota Tanaka, Hirofumi Jono, Hiroki Itoh, Daisuke Kadowaki, Koki Tokunaga, Akitomo Shibata, Harumi Tanoue, Kazuya Ichikado, Ayami Kajiwara-Morita, Kentaro Oniki, Junji Saruwatari

**Affiliations:** 1 Department of Pharmacy, Saiseikai Kumamoto Hospital, 5-3-1 Chikami, Minami-ku, Kumamoto 861-4193, Japan; 2 Clinical Research Center, Saiseikai Kumamoto Hospital, 5-3-1 Chikami, Minami-ku, Kumamoto 861-4193, Japan; 3 Division of Pharmacology and Therapeutics, Graduate School of Pharmaceutical Sciences, Kumamoto University, 5-1 Oe-honmachi, Chuo-ku, Kumamoto 862-0973, Japan; 4 Department of Psychiatry, School of Medicine, Dokkyo Medical University, 880 Kitakobayashi, Mibu, Shimotsugagun 321-0293, Tochigi, Japan; 5 Department of Pharmacy, Kumamoto University Hospital, 1-1-1 Honjo, Chuo-ku, Kumamoto 860-8556, Japan; 6 Department of Clinical Pharmacy, Oita University Hospital, 1-1 Idaigaoka, Hasama-machi, Yufu 879-5593, Oita, Japan; 7 Department of Clinical Pharmaceutical Sciences, Graduate School of Pharmaceutical Sciences, Kumamoto University, Kumamoto 862-0973, Japan; 8 Faculty of Pharmaceutical Sciences, Sojo University, 4-22-1 Ikeda, Nishi-ku, Kumamoto 860-0082, Japan; 9 Division of Respiratory Medicine, Saiseikai Kumamoto Hospital, 5-3-1 Chikami, Minami-ku, Kumamoto 861-4193, Japan

**Keywords:** cancer chemotherapy, neutropenia, nonlinear mixed-effects model, pharmacometrics, physiologically based pharmacokinetic model

## Abstract

**Background/Objectives:** Pemetrexed–platinum chemotherapy is a key treatment option for non-squamous non-small cell lung cancer (NSCLC); however, its use is often limited by hematologic toxicity, particularly neutropenia. We aimed to develop a model-informed framework for assessing neutrophil dynamics using routinely available clinical data and pemetrexed exposure. **Methods:** This real-world investigation included 86 patients with NSCLC who received pemetrexed–platinum chemotherapy for model development, and 83 patients who received the same chemotherapy plus pembrolizumab or bevacizumab for validation. We developed a nonlinear mixed-effects model to predict neutrophil dynamics during the first cycle following pemetrexed–platinum chemotherapy, using patient-specific clinical data collected before chemotherapy initiation and pemetrexed pharmacokinetic parameters derived from physiologically based pharmacokinetic (PBPK) modeling. **Results:** The final model suggested that the area under the curve (AUC)_0–24_ >175 μg·h/mL for pemetrexed, blood urea nitrogen, and concomitant use of renin–angiotensin system inhibitors influenced neutrophil suppression and delayed recovery. The receiver operating characteristic curve (AUROC) for identifying patients with a neutrophil count <1500/μL immediately before the anticipated next treatment cycle was 0.768 (95% CI: 0.639–0.898) in the development cohort, and 0.718 (95% CI: 0.545–0.891) in the validation cohort. **Conclusions:** This model-informed framework, based on PBPK-derived pemetrexed exposure and routinely available clinical factors, may help identify patients at risk of clinically relevant neutropenia that could delay the initiation of the next treatment cycle.

## 1. Introduction

Lung cancer was the most frequently diagnosed malignancy worldwide in 2022, with approximately 2.5 million new cases, representing 12.4% of all cancer diagnoses [1]. It also remains the leading cause of cancer-related death, with an estimated 1.8 million deaths, accounting for 18.7% of global cancer mortality [1]. Recent advances in diagnostic and therapeutic approaches have improved survival outcomes for patients with lung cancer [2]. For advanced non-small cell lung cancer (NSCLC) lacking driver gene mutations (approximately 50% of cases), the standard first-line treatment is either an immune checkpoint inhibitor (ICI) monotherapy or a combination of ICI and platinum-based chemotherapy [3,4]. In a phase III study, pemetrexed plus cisplatin demonstrated noninferiority in overall survival (OS) compared with gemcitabine plus cisplatin in untreated advanced NSCLC [5]. Subgroup analyses further showed that pemetrexed significantly improved survival among patients with adenocarcinoma and large-cell carcinoma, indicating its selective efficacy in non-squamous histology [5]. Furthermore, pemetrexed-containing regimens were associated with lower rates of hematologic and gastrointestinal toxicities than those containing gemcitabine [5]. These favorable efficacy and safety profiles establish pemetrexed as a key treatment option for non-squamous NSCLC [3].

Nevertheless, hematological toxicity, particularly neutropenia, is the most common adverse event associated with pemetrexed–platinum chemotherapy [3,5]. Neutropenia is clinically significant because it markedly increases the risk of infection. These infections can reduce patients’ quality of life and, in severe cases, become life-threatening [6]. To prevent such infections, clinicians often delay or withhold subsequent chemotherapy cycles when neutropenia occurs. However, these treatment delays and prolonged intervals between chemotherapy cycles in first-line NSCLC treatment have been associated with poorer survival outcomes [7]. Therefore, early prediction of neutropenia is crucial for maintaining treatment continuity, which is essential for improving survival outcomes and preventing infections in patients with NSCLC.

Platinum-based agents, such as cisplatin and carboplatin, are widely used in combination chemotherapy, but their dosing strategies differ based on renal function [8]. Cisplatin is generally administered at a fixed dose based on body surface area, although it is often avoided or dose-reduced in patients with impaired renal function (e.g., GFR < 60 mL/min) due to its nephrotoxicity [9]. In contrast, carboplatin clearance is strongly correlated with glomerular filtration rate (GFR), and a linear relationship has been demonstrated between the area under the concentration–time curve (AUC) and carboplatin dose [8,10]. Based on these pharmacokinetic (PK) characteristics, Calvert et al. developed a formula to calculate the appropriate carboplatin dose from the patient’s measured GFR and target AUC [10]. On the other hand, no standardized dose adjustment guidelines currently exist for pemetrexed, despite its primary renal excretion [8]. Whether renal function assessed by creatinine clearance (CLcr) or estimated GFR (eGFR) can predict pemetrexed-induced neutropenia remains controversial [11,12,13]. A population pharmacokinetic analysis by Latz et al. demonstrated that pemetrexed clearance is significantly influenced by renal function and that increased systemic exposure (AUC) is associated with a higher risk of neutropenia, suggesting the potential clinical relevance of renal function-based dose considerations [14]. However, a recent study demonstrated that dose-adjusted pemetrexed can be safely used in patients with renal impairment, indicating that effective treatment may be feasible even in this traditionally excluded population [15]. Therefore, appropriate dose adjustment according to renal function is essential. Studies have reported a significant association between total systemic exposure to pemetrexed and an increased incidence of severe hematological adverse events [16,17]. Based on the above information, considerable interindividual variability in the risk of pemetrexed-induced neutropenia may be influenced by patient-specific clinical and PK characteristics. However, blood pemetrexed levels are not routinely measured in clinical practice, which complicates the calculation of PK parameters.

Several semi-mechanistic population PK/pharmacodynamic (PD) models have previously characterized pemetrexed-induced neutropenia in detail. For example, Latz et al. developed an integrated PK/PD model incorporating a physiological description of hematopoiesis within a nonlinear mixed-effects (NLME) framework [18]. In contrast to cumulative exposure metrics such as AUC, Boosman et al. suggested that pemetrexed-related toxicity may be better characterized by a threshold exposure metric defined by the duration for which plasma concentrations exceed the toxicity threshold, rather than by total systemic exposure [19]. These semi-mechanistic approaches provide biologically grounded insights into exposure–toxicity relationships and allow for simultaneous estimation of PK and PD parameters. Although these models were developed using well-controlled clinical trial datasets and provide valuable mechanistic insights, real-world clinical practice involves patients with heterogeneous backgrounds, comorbidities, and variable renal function, often without therapeutic drug monitoring. Furthermore, regimens combining platinum agents with pemetrexed, as well as the addition of ICIs, have become increasingly common, and the ongoing evolution of combination therapies in oncology further complicates the effects of treatment on neutrophil dynamics in real-world clinical practice. Such variability may result in substantial interindividual differences in neutrophil dynamics, underscoring the need for modeling strategies that remain applicable in routine clinical settings.

Physiologically based PK (PBPK) modeling describes and predicts a drug’s behavior across various physiological tissues [20]. Meanwhile, NLME modeling is widely used not only in PK and PK/PD analyses but also in longitudinal clinical laboratory data modeling [21,22]. It enables the evaluation of various structural models and covariate effects, uses all available observation points, and facilitates model validation through simulation. These approaches provide a framework for predicting treatment-related toxicities using routinely available clinical data, thereby accommodating the substantial variability encountered in real-world clinical practice. In heterogeneous treatment settings such as combination chemotherapy, flexible modeling approaches may also help identify novel and/or clinically relevant factors associated with toxicity and provide a basis for future refinement toward more mechanistic modeling strategies.

The primary objective of this study was to develop a model-informed framework for assessing neutrophil dynamics during the first cycle of pemetrexed–platinum chemotherapy from routinely available clinical data, without relying on measured plasma pemetrexed concentration, by constructing a population model of neutrophil dynamics using an NLME modeling approach. In this study, we focused on the first treatment cycle, which allows prediction under conditions in which empirical dosing is applied without prior dose adjustment or supportive interventions, thereby enabling assessment of baseline patient-specific risk. In addition, we aimed to identify patient-specific risk factors, including patient characteristics from medical records and pemetrexed PK parameters derived from a PBPK model, that influence changes in neutrophil counts during the first cycle of pemetrexed–platinum chemotherapy in patients with lung cancer. Furthermore, we validated whether the developed model is generalizable to other patients receiving combination regimens that include ICIs or molecularly targeted agents.

## 2. Materials and Methods

### 2.1. Study Population

In this retrospective study, we analyzed data from two patient cohorts at Saiseikai Kumamoto Hospital from January 2015 to December 2023. The detailed inclusion and exclusion criteria for the modeling dataset were as follows: (1) received pemetrexed–platinum chemotherapy for at least one cycle; (2) had accurate records of treatment initiation and discontinuation; (3) underwent at least one hematological examination during the first treatment cycle; (4) had no prior exposure to pemetrexed or platinum agents; and (5) provided written informed consent. The development cohort comprised 86 patients with NSCLC (62 men and 24 women; mean age, 68.24 ± 7.85 years) who received pemetrexed–platinum therapy, including 38 patients treated with cisplatin plus pemetrexed and 48 patients treated with carboplatin plus pemetrexed. To evaluate the applicability of the model to regimens combined with antibody drugs, such as pembrolizumab or bevacizumab, which are currently used as standard treatments, the validation cohort included 83 patients with NSCLC (51 men and 32 women, 65.52 ± 7.04 years), comprising 39 patients treated with carboplatin plus pemetrexed plus pembrolizumab, 20 with cisplatin plus pemetrexed plus bevacizumab, and 24 with carboplatin plus pemetrexed plus bevacizumab. In all regimens, chemotherapeutic agents and antibody drugs were administered on day 1 of the 21-day cycle. The standard doses of the chemotherapeutic agents were as follows: 500 mg/m^2^ of pemetrexed, 75 mg/m^2^ of cisplatin, and multiplying an AUC of 5 mg·min/mL by [25 + GFR (mL/min)] for carboplatin (mg); however, the actual doses of pemetrexed and platinum agents were determined empirically by physicians based on clinical judgment, taking into account patient-specific factors such as age, renal function, performance status, and comorbidities. For the validation cohort, pembrolizumab was administered at a fixed dose of 200 mg, and bevacizumab at 15 mg/kg, according to standard clinical protocols at the time. Neutrophil data from all measurement points in the first treatment cycle were used in the analysis, excluding any time points after the administration of granulocyte colony-stimulating factor. Patients with only a single hematological observation were included in the analysis and treated the same as those with multiple observations. No additional weighting based on the number of observations per patient was applied, consistent with the NLME modeling framework, in which all available data contribute to estimating population parameters. The final datasets comprised 316 and 375 data points from the development and validation cohorts, respectively, after excluding 6 data points (from 3 patients) and 5 data points (from 1 patient), respectively. The following information was collected from patient medical records: sex, age, height, weight, body mass index (BMI), creatinine, blood urea nitrogen (BUN), chemotherapy regimens, doses of anticancer agents, and other concomitant medications. Renal function was estimated using the Cockcroft–Gault formula for CLcr and the Japanese equation for individualized eGFR [23]. The eGFR values were de-indexed from 1.73 m^2^ to each patient’s body surface area (BSA), calculated using the Du Bois formula, to obtain absolute values (mL/min).

### 2.2. Developing a PBPK Model for Pemetrexed

A PBPK model for pemetrexed was developed using PK-Sim (version 11.3, Open Systems Pharmacology Suite). Physicochemical and PK parameters (including molecular weight, logP, pKa, plasma protein binding, and renal clearance) were obtained from the literature [24] and the PubChem database [25] (Supplementary Table S1). Model parameters not directly available were optimized using the Levenberg–Marquardt algorithm implemented in PK-Sim. For parameter optimization, pharmacokinetic summary metrics [clearance (CL), maximum plasma concentration (C_max_), and AUC from time zero to infinity (AUC_0–inf_)] derived from a single phase I study conducted on Japanese patients [26], in which pemetrexed was administered at a dose of 500 mg/m^2^, were used. The GFR fraction was adjusted to reproduce the observed values using the Levenberg–Marquardt algorithm implemented in PK-Sim. A single-study calibration approach was adopted to ensure internal consistency of the optimization dataset and to avoid introducing heterogeneity across studies. Parameter estimation was therefore not performed using pooled data from multiple studies, and no separate parameter sets were optimized for individual studies. The parameters primarily optimized were those related to renal clearance and distribution.

Pemetrexed is a hydrophilic antifolate primarily eliminated by renal clearance (~70–90%), with limited hepatic metabolism [24]. The renal excretion process was modeled to account for glomerular filtration and active secretion, reflecting the known involvement of transporters. The PBPK simulations were conducted assuming pemetrexed monotherapy, and potential drug–drug interactions with concomitant therapies were not explicitly incorporated.

### 2.3. Evaluation of the PBPK Model for Pemetrexed

For model evaluation, independent datasets were used to ensure that model performance was assessed separately from the parameter optimization process. Virtual populations based on Nakagawa K et al. [26] and Kavathiya K et al. [27] were used to evaluate predictive performance across a range of doses, while data from Mita AC et al. [28] were used to assess model performance across different levels of renal function. Model performance was evaluated by comparing predicted and observed values [26,27,28] for CL, C_max_, and AUC_0–inf_. All PBPK simulations were conducted assuming pemetrexed monotherapy, and potential drug–drug interactions associated with combination regimens were not explicitly incorporated. Predicted values for these PK parameters were obtained through simulations in which pemetrexed was administered to virtual populations generated in PK-Sim. In creating virtual populations, the number of individuals, race, proportion of females, and age (mean and range) were set to match those reported in the literature [26,27,28]. Model performance was assessed using the average fold error (AFE) between predicted and observed PK parameters. AFE was calculated using Equation (1):

AFE = Predicted value/Observed value(1)

A two-fold error range (AFE between 0.5 and 2) was considered acceptable for model evaluation [29].

First, the predictive performance of the PBPK model was evaluated without accounting for differences in varying renal function. Predicted PK parameters were obtained for eight pemetrexed doses (300, 500, 600, 700, 800, 900, 1000 and 1200 mg/m^2^) for which observed values were reported in the literature [26,27]. These PK parameters were predicted from simulations in healthy adult populations, as patients with impaired renal or hepatic function were excluded from the studies that reported the observed data [26,27]. To evaluate model performance, AFE was calculated for each PK parameter at each dose simulation, and the geometric mean of the AFEs was subsequently computed.

Subsequently, model performance was assessed across populations with varying renal function by comparing predicted and observed [28] PK parameters for three groups: GFR ≥ 80, 60–79, and 40–59 mL/min. The mean GFR for each group was set to match previously reported values [28]. PK parameters for the GFR 40–59 mL/min group were predicted using simulations of a chronic kidney disease population, whereas those for the other groups were predicted using simulations of healthy adult populations. The AFE for the PK parameters was calculated for each group, and the predicted plasma concentration–time profiles (arithmetic mean and 5th–95th percentiles) were visually compared with the observed data [28] for each GFR group. Observed concentration–time data [28] were digitized from the published figures using WebPlotDigitizer (version 4.8, Ankit Rohatgi, Pacifica, CA, USA). Plasma concentration–time profiles were illustrated using R (version 4.1.1, R Foundation for Statistical Computing, Vienna, Austria).

### 2.4. Developing a Population Model for Changes in Neutrophil Counts over Time

NONMEM (version 7.5.1, ICON Dev Soln, Ellicott City, MD, USA) was used to model changes in neutrophil count during the first cycle of pemetrexed–platinum therapy. The model was fitted to neutrophil data using the first-order conditional estimation with interaction method. Changes in neutrophil count during the first cycle were best described visually by a quadratic function. The base model comprised two quadratic functions: one for the decrease (Equation (2)) and the other for the recovery (Equation (3)) of the neutrophil count.

y = A1 × (WEEK − B)^2^ + Base − A1 × B^2^ × exp(ε) if WEEK is lower than B (2)

y = A2 × (WEEK − B)^2^ + Base − A1 × B^2^ × exp(ε) if WEEK is greater than B (3)

WEEK is the elapsed time in weeks, calculated as the number of days since pemetrexed administration divided by seven. Base denotes neutrophil counts before pemetrexed administration (typically within 1 week). A1 and A2 represent the slopes of neutrophil decline and recovery, respectively. B is the WEEK when neutrophil recovery starts, and ε denotes intra-individual variability with mean zero and variance σ^2^. To identify factors influencing interindividual variability in neutrophil counts following pemetrexed and platinum administration, the effects of various covariates on parameters A1, A2, and B were assessed.

**Step 1:** A model was constructed using routinely available clinical variables in standard medical practice. The following covariates were evaluated: age, BMI, BSA, renal function parameters (CLcr, eGFR, and BUN), pemetrexed dose, type of platinum-based agent (cisplatin or carboplatin), and the use of concomitant medications, including non-steroidal anti-inflammatory drugs, renin–angiotensin system (RAS) inhibitors, and diuretics. Covariate values were obtained at the initiation of chemotherapy during the first cycle.

**Step 2:** In the second step, instead of the pemetrexed dose, PBPK model-derived PK parameters of pemetrexed (CL, C_max_, AUC_0–inf_, and AUC from time zero to 24 h [AUC_0–24_]) were included as covariates to capture interindividual differences in drug exposure beyond dose. In this framework, PBPK-derived exposure metrics were incorporated as baseline covariates rather than time-varying inputs, given the primary objective of developing a parsimonious and robust predictive model applicable to routine clinical data settings where longitudinal PK information is typically unavailable. Both AUC_0–24_ and AUC_0–inf_ were evaluated as potential exposure metrics in continuous and categorical forms during covariate selection, and candidate cut-off values for categorical covariates were empirically explored based on model performance.

The covariate model was developed using univariate analysis in NONMEM, followed by stepwise selection with forward inclusion and backward elimination. The effect of covariates on parameters A1, A2 and B was assessed using the likelihood ratio test, wherein changes in the objective function value (OFV) computed by NONMEM were compared against critical values from the chi-squared (χ^2^) distribution. In the forward step, covariates that exhibited significant effects in the univariate analysis were added to the model in descending order of their impact on OFV. The covariates were included in the model when the OFV decreased by >3.84 (χ^2^, *p* < 0.05, one degree of freedom) and >5.99 (χ^2^, *p* < 0.05, two degrees of freedom), thereby developing the full model. In the backward step, models with only one covariate excluded from the full model were developed. The covariates were retained in the model when the OFV increased by >3.84 (χ^2^, *p* < 0.05, one degree of freedom) and >5.99 (χ^2^, *p* < 0.05, two degrees of freedom), resulting in the final model.

### 2.5. Model Evaluation of the Development Cohort

The model evaluation was performed using the same procedures previously reported [30]. The final model was internally validated using goodness-of-fit (GOF) plots and prediction-corrected visual predictive checks (pcVPCs). A stratified nonparametric bootstrap analysis was also performed to investigate model robustness and parameter precision. The GOF plots used scatter plots of observed versus population-predicted values, observed versus individual-predicted values, and conditional weighted residuals versus population-predicted values and time after the pemetrexed dose. A total of 1000 bootstrap replicates were performed to assess parameter uncertainty. This number was selected based on common practice in pharmacometric analyses, providing a balance between computational efficiency and stability of confidence interval estimation. In the pcVPC approach, the 5th, 50th and 95th percentiles of simulations from the final model were compared with the observed values. The 95% confidence intervals (CIs) for the parameters after successful bootstrap convergence were compared with the final model estimates. In addition, receiver operating characteristic (ROC) analysis was performed based on population-predicted neutrophil counts (PRED) obtained from the final model. The analysis was performed at the patient level, with each patient contributing one outcome. The outcome was defined as a neutrophil count <1500/μL immediately before the anticipated next treatment cycle (days 21–28). We used the neutrophil count immediately before the anticipated next cycle because the model we developed covers both the decline and recovery phases of the neutrophil counts, and because, in clinical practice, the value immediately before the anticipated next cycle is important for determining whether to reduce the dose or suspend treatment. For each patient, the neutrophil count predicted by the final model at the corresponding time point was used to assess discrimination. The ROC curve was constructed to evaluate the model’s ability to discriminate between observations below and above this threshold, and predictive performance was assessed using the area under the ROC curve (AUROC).

### 2.6. Final Model Verification in Validation Cohort

To evaluate the predictive performance and generalization ability of the final model, we conducted an evaluation using data from the validation cohort. PK parameters of pemetrexed—specifically CL, C_max_, AUC_0–inf_ and AUC_0–24_—were simulated for each patient in the validation cohort using the PBPK model constructed in the development cohort. Subsequently, individual neutrophil counts during the first treatment cycle were predicted using Bayesian estimation based on the final model implemented in NONMEM. Model performance was assessed using GOF plots and the pcVPC approach, following the same procedure as in the model validation of the development cohort. Furthermore, an ROC curve was generated to assess the ability of the final model to discriminate patients with a neutrophil count <1500/μL immediately before the anticipated next treatment cycle (days 21–28), and its discriminative ability was assessed using the AUROC. Statistical analyses and graphics were generated using R.

## 3. Results

### 3.1. Patient Demographics

A comparison of the demographic and clinical characteristics of patients in the development (n = 86) and validation (n = 83) cohorts is summarized in Table 1. Patients in the validation cohort received higher doses of pemetrexed, had a higher proportion of concomitant carboplatin use, were younger, and had higher BMI values than those in the development cohort. No significant differences were observed in other baseline characteristics.

### 3.2. PK Parameter Estimation Using the PBPK Approach

The demographic characteristics of the virtual populations used in the PBPK simulations are summarized in Supplementary Table S2 and are consistent with those reported in the corresponding clinical studies. Supplementary Table S3 summarizes the predicted PK parameters, the corresponding observed values reported in the [26,27], and the AFE for each pemetrexed dose. The developed PBPK model predicted PK parameters within a two-fold error range across the wide range of pemetrexed dose levels. Moreover, the geometric mean (95% CI) of the AFE for CL, C_max_, and AUC_0–inf_ were 1.04 (0.86–1.26), 0.91 (0.80–1.05), and 1.00 (0.84–1.20), respectively, suggesting favorable predictive performance. Model evaluation across three virtual populations with varying GFRs similarly showed PK predictions within a two-fold error range for each group (Supplementary Table S4). The developed PBPK model successfully captured the expected PK trend: as GFR declines, CL decreases, and AUC_0–inf_ increases, while C_max_ generally remains relatively unchanged [28]. In addition, simulated 24-h plasma concentration–time profiles of pemetrexed reasonably reproduced the overall exposure observed in clinical data [28]. However, discrepancies were observed during the early distribution phase (Supplementary Figure S1), supporting the validity of the developed PBPK model. Subsequently, individual CL, C_max_, AUC_0–inf_, and AUC_0–24_ values were calculated for each participant using the developed PBPK model and patient-specific information, including race (Japanese), age, sex, height, weight, BMI, BSA, eGFR, and pemetrexed dose. AUC_0–inf_ and AUC_0–24_ showed weak correlations with pemetrexed dose (r = 0.240 and r = 0.262, respectively) and moderate inverse correlations with eGFR (r = −0.652 and r = −0.637, respectively). AUC_0–inf_ and AUC_0–24_ were almost perfectly correlated (r = 0.999) (Supplementary Figure S2).

### 3.3. Development of the Final Model for the Change in Neutrophil Count

Supplementary Table S5 summarizes the impact of tested covariates on the OFV in the neutrophil count variation model. In Step 1, the model was developed using routinely available information and did not include PK parameters. The model parameters at this step were as follows:

A1: 0.717 × (Dose/760)^1.27^ × exp(η) (4)

A2: 0.0319 × exp(η)(5)

B: 1.32 × exp(η)(6)

where η represents interindividual variability with a mean of zero and variance ω^2^.

Step 1 yielded a predictive model that may explain changes in neutrophil counts solely by the pemetrexed dose. However, because we obtained real-world clinical data retrospectively, pemetrexed doses may have been adjusted based on individual patient characteristics, such as age and renal function. Furthermore, the model included only the pemetrexed dose, making it challenging to predict individualized dosing prior to chemotherapy initiation. Therefore, we incorporated the PK parameters for pemetrexed derived from the PBPK model (Step 2) to capture interindividual variability in pharmacokinetics beyond that captured by empirically adjusted doses. This extended analysis identified BUN, concomitant use of RAS inhibitors, and pemetrexed AUC_0–24_ as statistically significant covariates (Supplementary Table S6). Although BMI showed a significant univariate association with A2, it was not retained in the final model because inclusion during forward selection did not improve model fit (ΔOFV = +0.261). The variance of interindividual variability was slightly reduced across all parameters from Step 1 to Step 2 (Supplementary Table S7). However, substantial interindividual variability remained. The final model equations incorporating these covariates were as follows:

A1: 0.334 × (BUN/15.2)^−0.975^ × exp(η) (7)

A2: 0.0443 × 0.0451^RAS inhibitor^ × exp(η) (8)

B: 1.39 × 1.44^AUC0–24>175^ × exp(η) (9)

where RAS inhibitor = 1 if RAS inhibitor is administered concomitantly, otherwise 0; AUC_0–24_ > 175 = 1 if pemetrexed AUC_0–24_ exceeds 175 μg·h/mL, otherwise 0; and η represents interindividual variability with a mean of zero and variance ω^2^.

**Figure 1:**
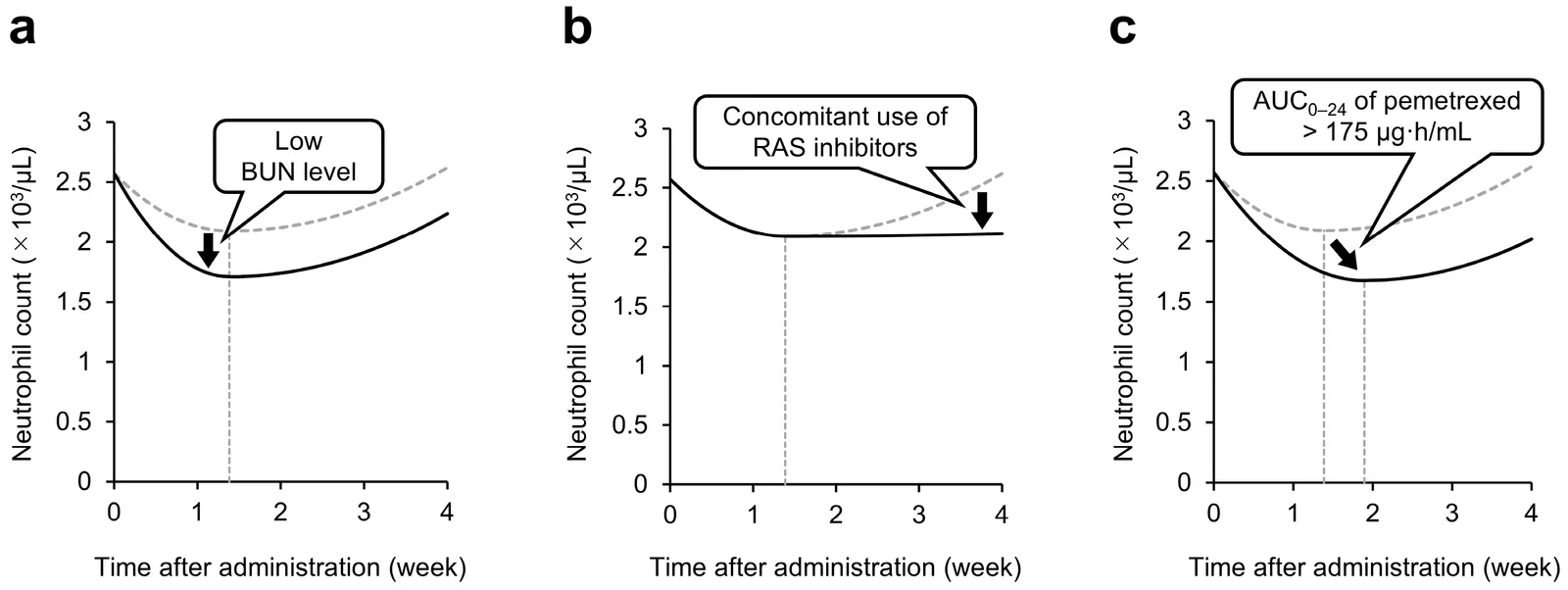
Relationship between the time after administration and neutrophil count based on the prediction model for neutropenia. The curves represent model-simulated neutrophil trajectories for illustrative purposes and are not observed clinical data. (**a**) Relationships between the time after administration and neutrophil count are represented as a solid curve for patients with a low BUN. They are presented as a dotted curve for average patients. (**b**) Relationships between the time after administration and neutrophil count are represented by a solid curve for patients taking a RAS inhibitor and are presented as a dotted curve for the average patient. (**c**) Relationships between the time after administration and neutrophil count are represented by a solid curve for patients with a high value of AUC_0–24_. They are presented as a dotted curve for average patients. AUC_0–24_, area under the plasma concentration–time curve from time zero to 24 h; BUN, blood urea nitrogen; RAS, renin–angiotensin system.

The effects of individual patient factors incorporated into the final model are illustrated in Figure 1. The model suggested that patients with lower BUN levels experienced a more rapid decline in neutrophil counts (Figure 1a), those receiving concomitant RAS inhibitors showed delayed recovery (Figure 1b), and patients with a pemetrexed AUC_0–24_ > 175 μg·h/mL exhibited a delayed onset of neutrophil recovery (Figure 1c).

We also evaluated the potential contribution of platinum treatment. Platinum type was tested as a covariate and was additionally forced into different components of the model in sensitivity analyses. The addition of platinum type resulted in only a small reduction in OFV when incorporated into the A1 component, whereas OFV increased when it was incorporated into the A2 or B components. Furthermore, the population predictions and objective function values were essentially unchanged compared with those of the original final model (Supplementary Figure S3 and Table S8). These findings suggest that platinum type did not materially improve the description or prediction of neutrophil dynamics in the present dataset.

### 3.4. Evaluation of the Final Model

**Figure 2:**
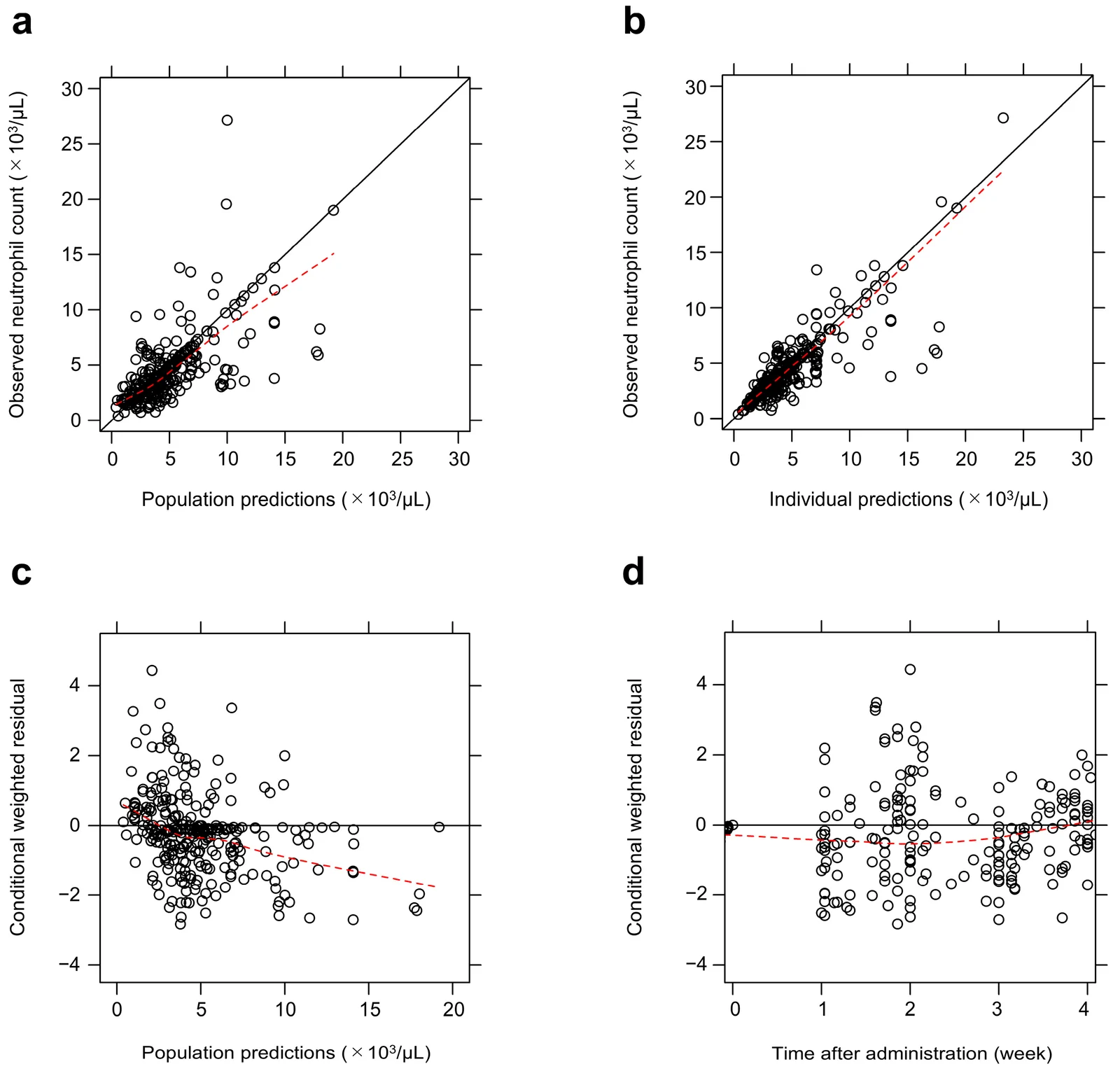
Goodness-of-fit plots for the final model in the development cohort. (**a**) Observed vs. population-predicted neutrophil count. (**b**) Observed vs. individual-predicted neutrophil count. (**c**) Conditional weighted residual vs. population-predicted neutrophil count. (**d**) Conditional weighted residual vs. time after administration. The solid lines in (**a**,**b**) represent the identity lines. The solid lines in (**c**,**d**) represent the zero line. The dashed lines represent smoothing lines.

**Figure 3:**
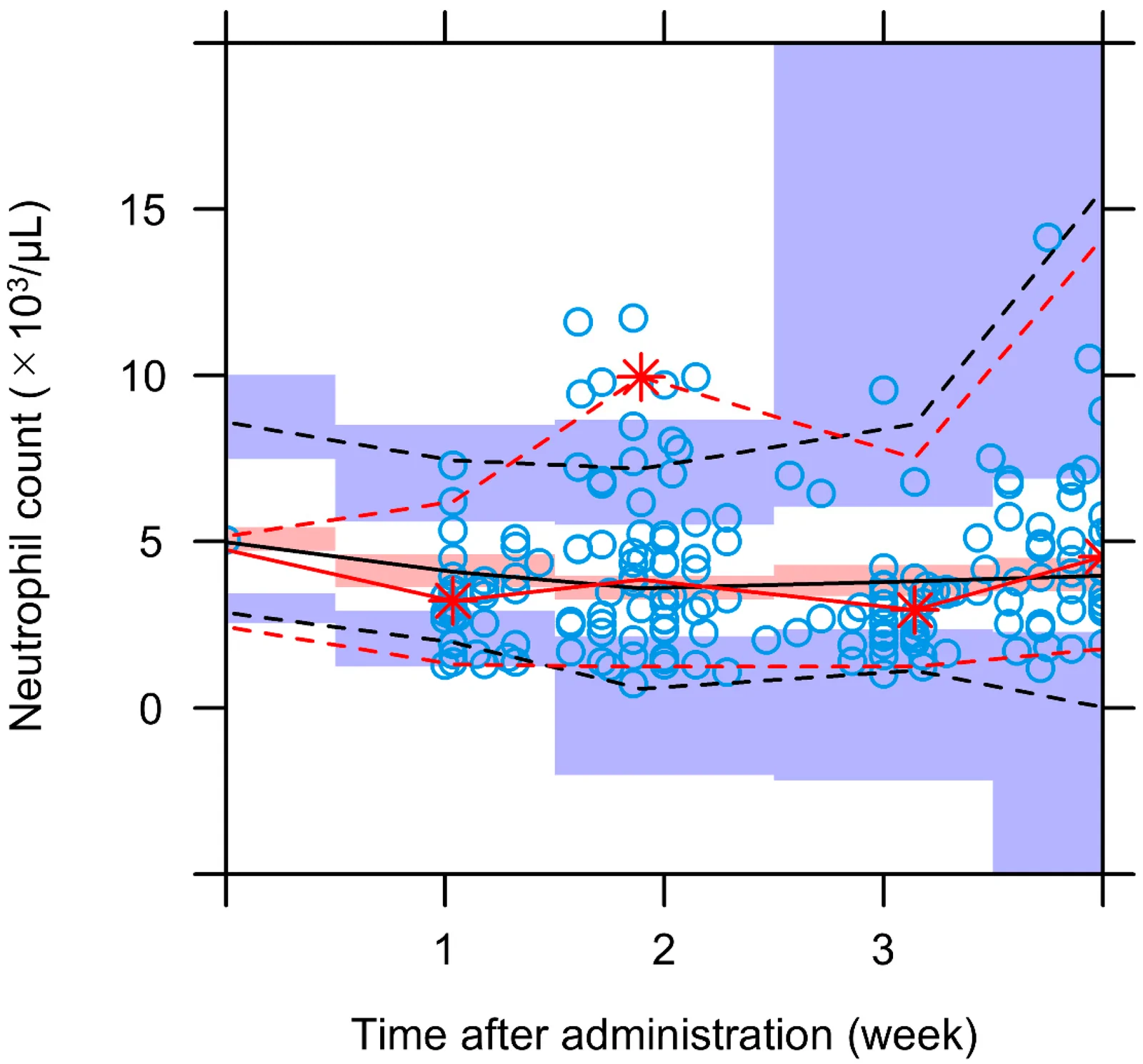
Prediction-corrected visual predictive check for the final model in the development cohort. Blue circles indicate observed neutrophil counts. The solid red line represents the median of the observed neutrophil count. The solid black line represents the median of the predicted neutrophil count in the simulated data. The dashed red lines are the 5th and 95th percentiles of the observed neutrophil count. The dashed black lines are the 5th and 95th percentiles of the predicted neutrophil count in the simulated data. The red area represents the 95% confidence interval for the median, while the blue area represents the 95% confidence interval for the 5th and 95th percentiles in the simulated data. The asterisk represents outliers at simulated timepoint; that is, the asterisk denotes the timepoint at which the solid and dashed red lines fall outside the 95% confidence intervals.

The GOF plots suggested a correlation between the neutrophil counts predicted by the final model and the actual measurements (Figure 2a,b). The distribution of residuals showed no significant pattern with respect to population-predicted values and time after pemetrexed administration (Figure 2c,d). The pcVPCs showed that the final model adequately described the overall observed neutrophil data (Figure 3). However, the model tended to underpredict the upper percentiles, while adequately capturing the median and lower percentiles relevant for the clinical assessment of neutropenia risk. Among 1000 bootstrap runs, 622 (62.2%) exhibited successful minimization and were included in the bootstrap analysis. The median parameter estimates obtained from the bootstrap analysis were consistent with those of the final model. The 95% CIs for all parameters obtained using the bootstrap approach were generally consistent with the NONMEM estimates. The 95% confidence intervals for key covariate effects did not cross 0 (for BUN on A1) and 1 (for RAS inhibitor on A2 and AUC_0–24_ on B), supporting the stability of the identified relationships (Supplementary Table S7). The relative standard errors (RSEs) exceeded 50% for the interindividual variability associated with parameters B, A2, and the effect of RAS inhibitor use on A2, indicating limited precision for these components. Although the inclusion of covariates reduced interindividual variability for several parameters, substantial unexplained variability remained. The η-shrinkage values were 15.9%, 49.8%, and 67.9% for the η components corresponding to ω_A1_, ω_A2_, and ω_B_, respectively, whereas ε-shrinkage was 11.0%. The relatively high η-shrinkage for ω_B_ indicates that individual estimates associated with this variability component should be interpreted cautiously. The final model used a diagonal covariance structure because the estimated correlations among the random effects associated with A1, A2, and B were weak. In an additional bootstrap-based assessment of covariate selection stability, AUC_0–24_, BUN, and RAS inhibitor use were selected in 102 (51%), 108 (54%), and 74 (37%) of 200 bootstrap replicates, respectively. Among the 86 patients in the development cohort, 10 (11.6%) had a neutrophil count <1500/μL immediately before the anticipated next treatment cycle (days 21–28), and the final model demonstrated moderate discriminative ability for this endpoint (AUROC, 0.768; 95% CI, 0.639–0.898). Sensitivity, specificity, positive predictive value (PPV), and negative predictive value (NPV) for this patient-level endpoint, together with their 95% CIs, are presented in Supplementary Table S9. Individual fits (observed vs. individual predictions over time) are provided in Supplementary Figure S4, demonstrating the model’s ability to capture neutrophil trajectories at the individual level.

### 3.5. Model Verification in Validation Cohort

**Figure 4:**
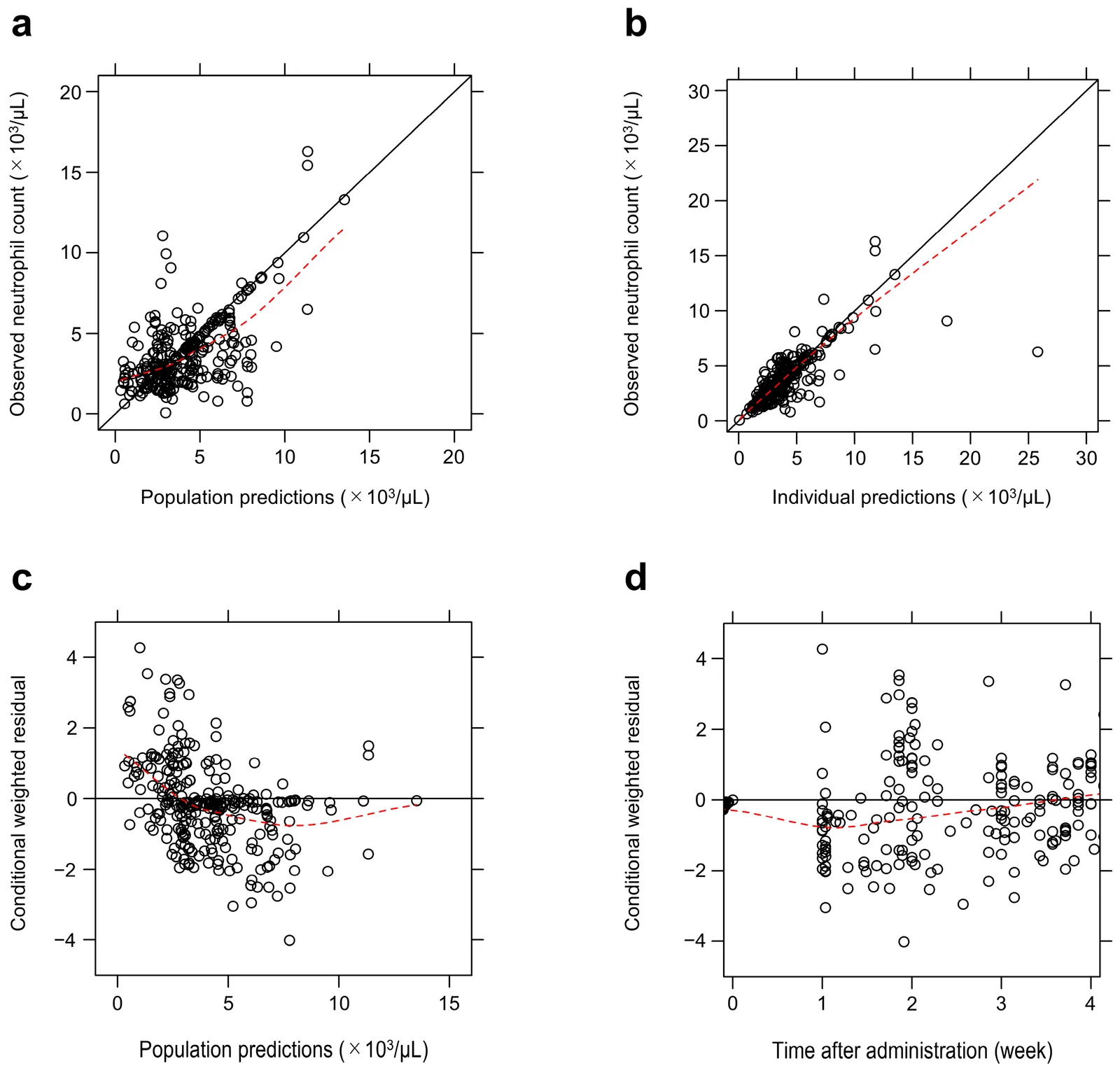
Goodness-of-fit plots for the final model in the validation cohort. (**a**) Observed vs. population-predicted neutrophil count. (**b**) Observed vs. individual-predicted neutrophil count. (**c**) Conditional weighted residual vs. population-predicted neutrophil count. (**d**) Conditional weighted residual vs. time after administration. The solid lines in (**a**,**b**) represent the identity lines. The solid lines in (**c**,**d**) represent the zero line. The dashed lines represent smoothing lines.

**Figure 5:**
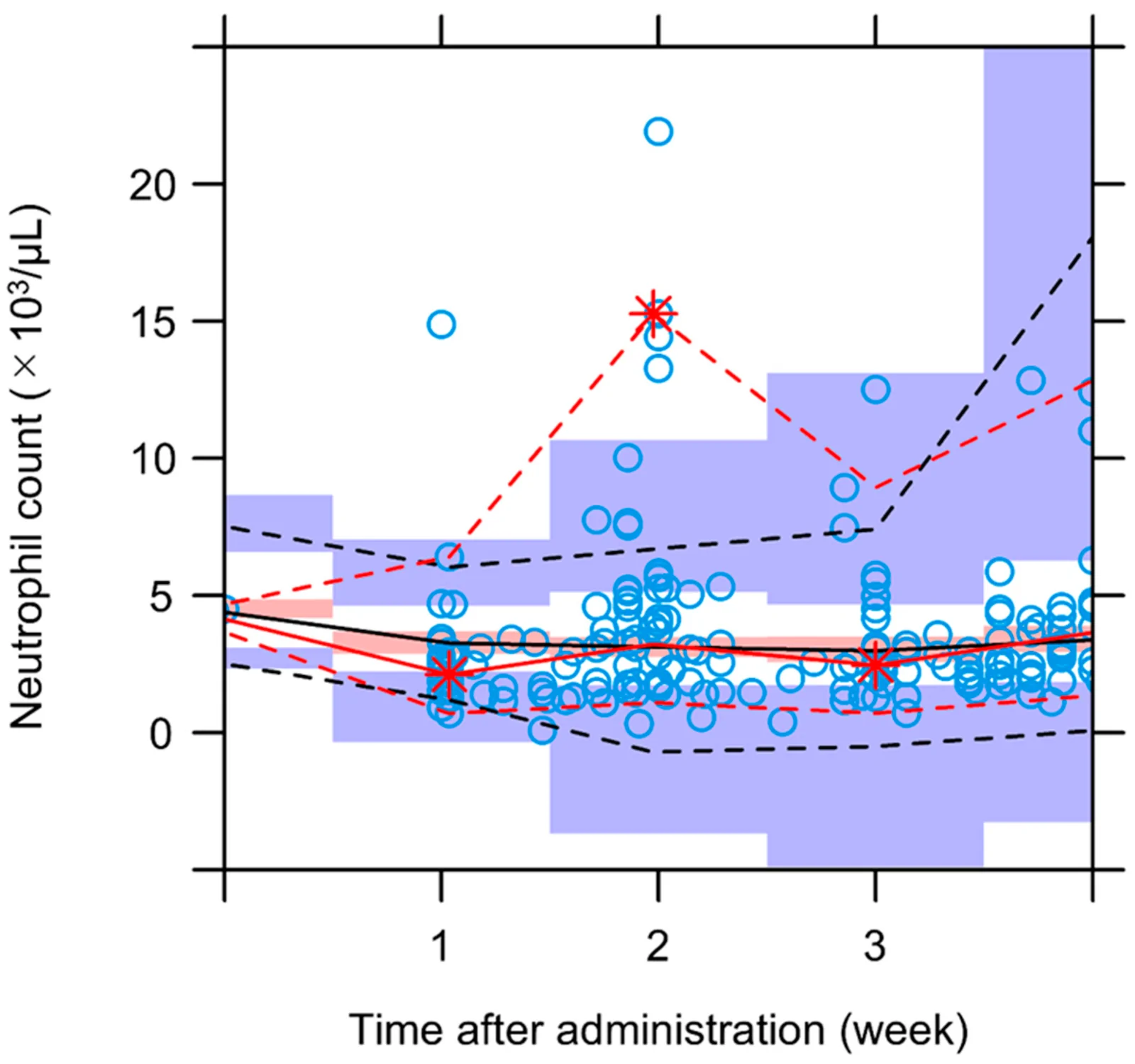
Prediction-corrected visual predictive check for the final model in the validation cohort. Blue circles plot the observed neutrophil counts. The solid red line represents the median of the observed neutrophil count. The solid black line represents the median of the predicted neutrophil count in the simulated data. The dashed red lines are the 5th and 95th percentiles of the observed neutrophil count. The dashed black lines are the 5th and 95th percentiles of the predicted neutrophil count in the simulated data. The red area represents the 95% confidence interval for the median, while the blue area represents the 95% confidence interval for the 5th and 95th percentiles in the simulated data. The asterisk represents outliers at simulated timepoint; that is, the asterisk denotes the timepoint at which the solid and dashed red lines fall outside the 95% confidence intervals.

Visual inspection of the GOF plots and pcVPCs confirmed that the final model accurately predicted changes in neutrophil counts among patients in the validation cohort, demonstrating performance comparable to that observed in the development cohort (Figure 4 and Figure 5). Among the 83 patients in the validation cohort, 5 (6.0%) had a neutrophil count <1500/μL immediately before the anticipated next treatment cycle (days 21–28), and the model demonstrated moderate discriminative ability for this endpoint (AUROC, 0.718; 95% CI, 0.545–0.891). Sensitivity, specificity, PPV, and NPV for this patient-level endpoint, together with their 95% CIs, are summarized in Supplementary Table S9.

## 4. Discussion

In this study, we developed a predictive model for neutrophil dynamics following pemetrexed–platinum chemotherapy using PBPK and NLME modeling approaches (Equations (7)–(9)). The model demonstrated moderate discriminative ability for identifying patients with a neutrophil count <1500/μL immediately before the anticipated next treatment cycle with an AUROC of 0.768 (95% CI: 0.639–0.898) in the development cohort. Furthermore, the validity of the final model was evaluated in a validation cohort comprising patients receiving combination therapy, including ICI and molecularly targeted drugs. Both cohorts were derived from the same institution and study period; therefore, independent multicenter validation is required to establish the model’s broader generalizability. Traditionally, the risk factors associated with neutropenia following pemetrexed–platinum therapy remain unclear, with dose adjustments often being made empirically. Our findings suggest the potential for data-driven, dose-individualized pemetrexed dosing based on quantitative predictions, even during the first treatment cycle, when neutrophil dynamics are not yet known, offering a path toward more personalized and safer chemotherapy regimens. In clinical practice, neutrophil counts at the start of the subsequent treatment cycle are particularly important for treatment decisions, such as dose modification or treatment delay. However, the present model captures neutrophil dynamics throughout the first treatment cycle. Therefore, the present model may provide clinically relevant information for anticipating neutrophil suppression before the next treatment cycle.

In the model constructed using only routinely available clinical data, pemetrexed dose was the only significant covariate (Equations (4)–(6)). However, the pemetrexed dose showed a weak but significant correlation with age and renal function (Supplementary Figure S5). For example, patients aged ≥75 years received significantly lower doses of pemetrexed than those aged <75 years (441.69 ± 74.51 mg/m^2^ vs. 477.44 ± 39.93 mg/m^2^, *p* = 0.007). These findings suggest that pemetrexed dosing was likely adjusted based on various patient characteristics, guided by clinical judgment and experience, as the data were collected retrospectively in a real-world setting. Therefore, the influence of different patient factors may have been indirectly reflected in the administered doses, which likely explains why only the dose was included in the initial model.

The neutrophil model developed in this study is empirical and has limited physiological interpretability, as it neither explicitly describes the underlying biological mechanisms nor directly links drug exposure to neutrophil kinetics. Although semi-mechanistic models of chemotherapy-induced myelosuppression are widely regarded as the gold standard for describing exposure–response relationships [31], the present study was designed to reflect the complexity of routine clinical practice, in which patients receive heterogeneous combination regimens and exhibit substantial variability in renal function, concomitant medications, prior treatments, and overall clinical condition. In particular, regimens combining platinum agents with pemetrexed, as well as the addition of ICIs, have become increasingly common, further complicating the interpretation of treatment effects on neutrophil dynamics. In such settings, flexible empirical modeling approaches may offer practical advantages by directly characterizing observed neutrophil trajectories without requiring strict mechanistic assumptions about the contributions of each treatment component. In addition to supporting clinically applicable prediction, this type of modeling may also help identify novel and/or clinically relevant covariates and temporal patterns associated with hematological toxicity in heterogeneous patient populations. Such findings may provide a useful basis for future refinement toward more mechanistic or semi-mechanistic modeling strategies as richer pharmacokinetic and longitudinal datasets become available. Therefore, we adopted an empirical modeling approach that directly characterizes neutrophil dynamics, prioritizing robustness and applicability in routine clinical settings over mechanistic interpretability. Given the ongoing evolution of combination therapies in oncology, such a flexible modeling framework may have broader applicability in future clinical settings where treatment regimens continue to diversify. In contrast to previous semi-mechanistic PK/PD models primarily developed using controlled clinical trial datasets [18,19], our approach was designed to accommodate the substantial variability encountered in real-world clinical practice.

By directly modeling neutrophil dynamics using routinely collected clinical data and PBPK-derived PK parameters, our framework enhances applicability in settings where therapeutic drug monitoring is unavailable. While several population pharmacokinetic (popPK) models of pemetrexed have been reported [32], their application generally requires observed concentration–time data or Bayesian estimation based on such data. In contrast, the PBPK approach enables estimation of individual exposure metrics solely from patient characteristics, making it more suitable for real-world clinical settings where pharmacokinetic sampling is not routinely performed. Furthermore, we investigated the influence of individual PK differences, beyond empirical dose adjustments, on neutrophil dynamics following pemetrexed–platinum chemotherapy. The final model demonstrated that a higher pemetrexed AUC_0–24_ was associated with a delayed onset of neutrophil recovery (Equation (9)), consistent with the semi-mechanistic physiological PD model of pemetrexed proposed by Latz et al., which quantitatively attributed delayed neutrophil recovery to prolonged maturation time of precursor cells as they transition from the maturation compartment to peripheral blood [16]. In high-exposure settings, the degree of precursor cell depletion increases, leading to a marked delay in peripheral blood recovery [16]. However, the differential effects of dose and exposure metrics identified in the model were derived empirically and should not be interpreted as reflecting distinct mechanistic pathways. Furthermore, exposure metrics were incorporated as baseline covariates rather than time-varying drivers, and therefore do not represent a fully mechanistic exposure–response relationship. In addition, longitudinal exposure metrics were not incorporated as time-varying drivers to maintain a parsimonious and robust model structure suitable for routine clinical data, where time-resolved exposure information is typically unavailable.

Previous work by Boosman et al. suggested that pemetrexed-related toxicity, particularly in patients with renal impairment, is more closely driven by a threshold exposure metric than by cumulative AUC [19]. In the present study, both AUC_0–24_ and AUC_0–inf_ were evaluated as potential covariates during model development. In the univariate analysis of candidate covariates, AUC_0–24_ showed a significant association with neutrophil dynamics, whereas AUC_0–inf_ did not. Importantly, AUC_0–24_ and AUC_0–inf_ were almost perfectly correlated (r = 0.999). This finding is consistent with the relatively short terminal half-life of pemetrexed (approximately 3 h) and its rapid renal elimination, indicating that AUC_0–24_ captures nearly the same overall exposure information as AUC_0–inf_ in the present setting. Therefore, it was presumed that the selection of AUC_0–24_ was not based on its mechanistically established or fundamentally superior status as a measure of exposure compared to AUC_0–inf_. Rather, it was selected empirically because, despite its near-equivalence to AUC_0–inf_ in terms of overall exposure, AUC_0–24_ showed a significant association with neutrophil dynamics in the present dataset. Therefore, the association between AUC_0–24_ and delayed neutrophil recovery observed in our study should be interpreted as a pragmatic exposure surrogate within our dataset rather than a definitive mechanistic driver of toxicity. We aimed to determine whether a parsimonious empirical exposure–response relationship could support clinically actionable risk prediction in settings where plasma concentration measurements are unavailable. Importantly, the exposure metrics in our model were simulated from patient characteristics without direct concentration measurements, supporting its applicability in real-world clinical settings.

Moreover, prolonged dosing intervals, potentially due to delayed neutrophil recovery, can increase mortality among patients receiving first-line treatment for NSCLC [7]. By leveraging PBPK and NLME modeling approaches, this study provides a novel framework for the detailed evaluation of exposure–toxicity relationships, suggesting the possibility of designing individualized dosing regimens that maximize efficacy and safety, even without plasma pemetrexed concentration measurements. Collectively, these findings emphasize the importance of optimizing pemetrexed dosing from the first treatment cycle onward to minimize the risk of severe toxicities, maintain the intended treatment schedule, and ultimately improve therapeutic outcomes.

In addition to drug exposure metrics, patient-specific factors such as BUN levels also influenced neutrophil dynamics (Equation (7)). This study demonstrated that the rate of neutrophil decline after pemetrexed administration was greater in patients with lower BUN levels. When participants were stratified by median BUN, the low-BUN group was younger, had better renal function, and had a higher rate of concomitant cisplatin use than the high-BUN group (Supplementary Table S10). Notably, no significant between-group differences in pemetrexed or platinum doses were observed, indicating that the observed decline in neutrophils was not attributable to differences in chemotherapy intensity. Given that cisplatin is generally associated with a lower frequency of severe neutropenia than carboplatin [33], the greater neutrophil decline observed in the low BUN group is unlikely to be explained solely by differences in chemotherapy regimens. Notably, younger age and preserved renal function are typically considered protective against severe myelosuppression [12,13,34]. Therefore, this finding appears counterintuitive and suggests the involvement of other underlying factors. BUN may have been incorporated into the model not solely as a marker of renal clearance, but also as a surrogate for broader physiological conditions—such as nutritional status or metabolic reserve—that influence hematologic sensitivity [34,35]. Thus, the observed association between low BUN and greater neutrophil decline may reflect a complex interplay of the aforementioned patient-specific factors. The observed association between lower BUN levels and greater neutrophil decline should be interpreted with caution. This finding is exploratory and may be influenced by unmeasured or uncontrolled confounding factors. For example, treatment selection may differ according to renal function and overall patient condition. Patients with lower BUN levels may have been more likely to receive cisplatin, whereas those with higher BUN levels may have been more likely to receive carboplatin. Given that cisplatin is typically administered at a fixed dose based on body surface area, whereas carboplatin is dosed according to renal function, differences in platinum agent selection and dosing strategy may have contributed to the observed association with neutrophil decline. Therefore, the relationship between BUN and neutrophil dynamics should not be interpreted as causal, and further studies are needed to clarify the underlying mechanisms. Nevertheless, further studies are warranted to confirm these findings and elucidate the underlying mechanisms.

Further, this study suggests that neutrophil recovery after pemetrexed administration is slower in patients taking RAS inhibitors (Equation (8)). Angiotensin II reportedly regulates hematopoiesis in vivo at the level of stem cells [36]. Moreover, angiotensin II analogs reportedly promote hematopoiesis and facilitate the recovery of circulating cells [37]. Therefore, RAS inhibitors may disrupt the hematopoietic regulatory function of angiotensin II, leading to delayed neutrophil recovery following pemetrexed administration. In addition, some RAS inhibitors, such as olmesartan and valsartan, have been reported to inhibit organic anion transporter 3 (OAT3) [38,39], which is crucial for pemetrexed excretion. Concomitant use of OAT3 inhibitors increases pemetrexed AUC, suggesting that RAS inhibitors could impair pemetrexed elimination and potentially increase drug exposure and toxicity risk [40,41]. However, the detailed mechanisms underlying this interaction remain unclear and warrant further investigation.

Recent advances in NSCLC treatment have demonstrated that the addition of antibody drugs, such as bevacizumab or pembrolizumab, to pemetrexed–platinum regimens can enhance therapeutic efficacy [3,42,43]. Although the addition of antibody drugs can cause a range of side effects, their impact on hematologic toxicity is generally minimal [3,43]. Given the increasing clinical use of these combination therapies, we validated the developed model using a separate cohort of patients receiving pemetrexed–platinum chemotherapy in combination with these antibody drugs. The final model demonstrated the ability to predict neutrophil count fluctuations after pemetrexed–platinum administration, even in the presence of concomitant antibody drug use. Therefore, the results of this study may be applicable across a wide range of pemetrexed–platinum regimens. Notably, several baseline differences were observed between the development and validation cohorts, including younger age, larger body size, higher administered doses, and a greater proportion of carboplatin-based regimens in the validation cohort. From a clinical perspective, these differences may influence PK and the risk of hematologic toxicity. Despite these variations, the model demonstrated comparable predictive performance in the validation cohort, suggesting it is reasonably robust and applicable across a range of patient characteristics and treatment settings. The PBPK model reasonably captured overall exposure, as reflected by the agreement between simulated and reported AUC values. However, discrepancies were observed in the concentration–time profiles, with a tendency to underpredict C_max_ and overpredict concentrations during the early distribution phase (approximately 2–6 h post-dose). These findings suggest that the model does not fully capture the bicompartmental distribution characteristics of pemetrexed.

This study has some limitations. First, individual platinum exposure was not directly quantified in the present study. Neutropenia during pemetrexed–platinum chemotherapy may reflect the combined myelotoxic effects of pemetrexed and platinum agents. Although platinum type was evaluated during model development and was additionally examined in sensitivity analyses by forcing it into different model components, its inclusion did not materially improve the overall model fit or predictive performance. However, the lack of individual platinum exposure metrics means that residual confounding by platinum exposure cannot be excluded. Therefore, the association between PBPK-derived pemetrexed exposure and neutrophil dynamics should not be interpreted as an isolated causal effect of pemetrexed exposure independent of platinum exposure. Second, as blood samples for the PK analysis were not collected from our study participants, the predictive performance of the PBPK model could not be directly evaluated. The PBPK model was evaluated using published clinical data and virtual populations constructed to match reported demographic characteristics. Therefore, the validation does not fully reflect individual-level predictive performance in the present study population and should be interpreted with caution. Third, the selection of AUC_0–24_ and the threshold used for exposure categorization were empirical and dataset-dependent. Although AUC_0–24_ and AUC_0–inf_ were almost perfectly correlated in the present PBPK simulations, the present analysis does not establish the superiority of AUC_0–24_ over AUC_0–inf_ as a toxicity-relevant exposure metric. Larger studies should evaluate alternative exposure representations, including continuous AUC_0–inf_ and different exposure thresholds, to determine the most robust exposure metric for predicting neutrophil suppression. Fourth, the robustness of the final model should be interpreted cautiously. Although the median bootstrap parameter estimates were generally consistent with those of the final model, successful minimization was achieved in only 622 of 1000 bootstrap replicates (62.2%), and some model components showed limited precision and relatively high η-shrinkage. In addition, substantial interindividual variability remained despite the inclusion of significant covariates, suggesting that additional clinical or treatment-related factors influencing neutrophil dynamics were not fully captured. Therefore, the stability of the identified covariate effects and the overall robustness of the model require confirmation in larger and independent datasets. Fifth, this study did not assess the relationship between pemetrexed exposure and antitumor efficacy. Although the model focused on hematologic toxicity, evaluating exposure–efficacy relationships is also crucial for dosage optimization. Future studies should investigate whether pemetrexed exposure is correlated with clinical outcomes, such as response or survival. Finally, although this observational study was designed to reflect real-world clinical conditions, its findings may have limited generalizability because of the relatively small sample size, the single-institution setting, and the exclusive inclusion of Japanese patients. These factors may restrict the applicability of the results to broader, more diverse populations. In addition, although the discrimination analysis was performed using population predictions that did not incorporate post hoc individual random-effect estimates informed by first-cycle neutrophil measurements, the model was not prospectively evaluated before treatment initiation. Therefore, prospective validation is required to determine whether the model can accurately identify patients at risk of neutrophil suppression in real-world clinical practice.

It should be noted that the present analysis was based on first-cycle data and did not include prospective or forward-looking predictions. Therefore, the model’s clinical utility for decision-making should be interpreted with caution. Importantly, this model is not intended to directly support dose adjustment decisions, as no explicit decision rules or dosing algorithms were established in this study. The primary aim of this study was to develop a predictive framework to describe neutrophil dynamics during the first treatment cycle, rather than to establish a fully validated decision-support tool for dose individualization. Nevertheless, the model may provide a basis for early identification of patients at higher risk of severe neutropenia using routinely available clinical data. Specifically, baseline clinical variables (e.g., renal function, baseline neutrophil count, and concomitant medications) can be used to derive individual exposure metrics via the PBPK simulations, which are subsequently incorporated into the NLME framework to simulate patient-specific neutrophil trajectories during the first treatment cycle. For example, estimated neutrophil trajectories could help identify patients at risk of significant neutropenia before the next treatment cycle, potentially informing closer monitoring or supportive care strategies. Importantly, the exposure metrics used in this model were derived from PBPK simulations based on patient characteristics and do not require direct PK measurements. Further prospective validation and simulation studies are required to establish its role in clinical decision-making.

## 5. Conclusions

The proposed model-informed framework enables early assessment of the risk of clinically relevant neutropenia during the first cycle of pemetrexed–platinum chemotherapy without requiring measured plasma concentrations of pemetrexed. The framework integrates PBPK-derived exposure estimates with routinely available patient characteristics and clinical factors, including BUN and concomitant RAS inhibitor use. Our findings suggest that data-driven, individualized pemetrexed dosing may provide a basis for future individualized dosing strategies, starting with the first treatment cycle, by accounting for individual patient characteristics. Although further validation with actual PK data and consideration of concomitant drug effects are needed, these results highlight the promise of model-informed strategies to optimize pemetrexed dosing and improve therapeutic outcomes.

## Figures and Tables

**Table 1:** Participant clinical characteristics.

	Development Cohort (n = 86)	Validation Cohort (n = 83)	*p*-Value
Age (years)	68.24 ± 7.85	65.52 ± 7.04	0.019
Sex (male/female)	62 (72.1)/24 (27.9)	51 (61.4)/32 (38.6)	0.191
Height (cm)	161.77 ± 9.10	161.88 ± 8.62	0.939
Weight (kg)	58.65 ± 11.01	62.10 ± 13.09	0.065
BMI (kg/m^2^)	22.34 ± 3.50	23.54 ± 3.79	0.034
BSA (m^2^)	1.61 ± 0.18	1.65 ± 0.20	0.170
Creatinine (mg/dL)	0.76 ± 0.16	0.76 ± 0.18	0.985
CLcr (mL/min)	77.32 ± 24.38	82.93 ± 24.16	0.135
eGFR (mL/min)	71.09 ± 19.14	70.78 ± 16.16	0.908
BUN (mg/dL)	15.25 ± 5.16	14.44 ± 3.93	0.257
Pemetrexed dose (mg)	760.64 ± 127.05	802.64 ± 116.72	0.027
Pemetrexed dose per BSA (mg/m^2^)	469.54 ± 51.35	484.27 ± 30.68	0.026
Co-administration			
Cisplatin/Carboplatin	38 (44.2)/48 (55.8)	20 (24.1)/63 (75.9)	0.009
Bevacizumab/Pembrolizumab	–	44 (53.0)/39 (47.0)	–
NSAIDs	23 (26.7)	12 (14.5)	0.058
RAS inhibitor	25 (29.1)	17 (20.5)	0.216
Diuretics	8 (9.3)	5 (6.0)	0.566
Number of hematological observations			0.664
1	2 (2.33)	0 (0)	
2	10 (11.6)	0 (0)	
3	37 (43.0)	16 (19.3)	
≥4	37 (43.0)	67 (80.7)	

Data are presented as means ± standard deviations or proportions for categorical variables. BMI, body mass index; BSA, body surface area; BUN, blood urea nitrogen; CLcr, creatinine clearance; eGFR, estimated glomerular filtration rate; NSAIDs, non-steroidal anti-inflammatory drugs; RAS, renin–angiotensin system.

## Data Availability

The datasets generated and/or analyzed during the current study are not publicly available due to individual privacy but are available from the corresponding authors on reasonable request.

## Supplementary Materials

The following supporting information can be downloaded at: https://www.mdpi.com/article/10.3390/pharmaceutics18080946/s1, Table S1. Physicochemical and biochemical parameters used in the PBPK model.; Table S2. Sampling settings for virtual populations employed in the PBPK simulations.; Table S3. Evaluation of the predictive performance of the PBPK model without consideration for GFR levels.; Table S4. Evaluation of the predictive performance of the PBPK model with consideration for GFR levels.; Table S5. Effects of the tested covariates on the objective values in univariate analysis.; Table S6. Summary of changes in objective values in stepwise covariate modeling.; Table S7. Parameter estimates of the NLME models and bootstrap analysis.; Table S8. Changes in the objective function value after adding platinum type as a covariate to different model parameters.; Table S9. Discriminative performance of the final model for identifying patients with a neutrophil count <1500/μL at the end of the first treatment cycle.; Table S10. Participant clinical characteristics stratified by the serum BUN level.; Figure S1. PBPK model-predicted and observed plasma concentration–time profiles of pemetrexed in each GFR group.; Figure S2. Correlations of PBPK-derived AUC0-24 and AUC0-inf with pemetrexed dose and eGFR, and between the two exposure metrics.; Figure S3. Goodness-of-fit plots for the final model and models with platinum type forced into different model parameters.; Figure S4. Individual fits of the final model in the development cohort.; Figure S5. Correlations between pemetrexed dose and patient characteristics.; Text S1. NONMEM control stream for the final model
