# Supplementary material for: Risk Assessment of Neutropenia Based on Exposure Information Without Plasma Concentration Measurement in Pemetrexed–Platinum-Based Chemotherapy: A Modeling Approach Using Real-World Clinical Data
Source: Pharmaceutics. 2026 Jul 31;18(8):946. doi: 10.3390/pharmaceutics18080946 (PMC13516902; doi:10.3390/pharmaceutics18080946)
Supplement: Supplementary file 1 [file pharmaceutics-18-00946-s001.zip › pharmaceutics-4437304-supplementary.pdf]

Pharmaceutics

Supplementary Materials

Prediction of Neutropenia Risk Based on Exposure Information Without Plasma Concentration Measurement in Pemetrexed–Platinum–Based Chemotherapy: A Modeling Approach Using Real-World Clinical Data.

Kazunori Morita[1,2,*], Keiichi Shigetome[3,4,*], Haruka Narise[3], Tetsuya Kaneko[3,5], Naoto Soejima[3], Ryota Tanaka[6], Hirofumi Jono[5,7], Hiroki Itoh[6], Daisuke Kadowaki[8], Koki Tokunaga[1], Akitomo Shibata[1], Harumi Tanoue[1], Kazuya Ichikado[2,9], Ayami Kajiwara-Morita[3], Kentaro Oniki[3], Junji Saruwatari[3,†]

1. Department of Pharmacy, Saiseikai Kumamoto Hospital, Kumamoto, Japan
2. Clinical Research Center, Saiseikai Kumamoto Hospital, Kumamoto, Japan
3. Division of Pharmacology and Therapeutics, Graduate School of Pharmaceutical Sciences, Kumamoto University, Kumamoto, Japan
4. Department of Psychiatry, School of Medicine, Dokkyo Medical University, Tochigi, Japan
5. Department of Pharmacy, Kumamoto University Hospital, Kumamoto, Japan
6. Department of Clinical Pharmacy, Oita University Hospital, Oita, Japan
7. Department of Clinical Pharmaceutical Sciences, Graduate School of Pharmaceutical Sciences, Kumamoto University, Kumamoto, Japan
8. Faculty of Pharmaceutical Sciences, Sojo University, Kumamoto, Japan
9. Division of Respiratory Medicine, Saiseikai Kumamoto Hospital, Kumamoto, Japan.

* These authors contributed equally to this work.

†Corresponding author:
Junji Saruwatari, Ph.D.
Division of Pharmacology & Therapeutics, Graduate School of Pharmaceutical Sciences, Kumamoto University, 5-1 Oe-honmachi, Chuo-ku, Kumamoto 862-0973, Japan
E-mail: junsaru@gpo.kumamoto-u.ac.jp

Contents

Supplementary Table S1. Physicochemical and biochemical parameters used in the PBPK model ········ 3

Supplementary Table S2. Sampling settings for virtual populations employed in the PBPK simulations ········ 4

Supplementary Table S3. Evaluation of the predictive performance of the PBPK model without consideration for GFR levels ········ 5

Supplementary Table S4. Evaluation of the predictive performance of the PBPK model with consideration for GFR levels ········ 7

Supplementary Table S5. Effects of the tested covariates on the objective values in univariate analysis ········ 9

Supplementary Table S6. Summary of changes in objective values in stepwise covariate modeling ········ 14

Supplementary Table S7. Parameter estimates of the NLME models and bootstrap analysis 15

Supplementary Table S8. Changes in the objective function value after adding platinum type as a covariate to different model parameters ········ 16

Supplementary Table S9. Discriminative performance of the final model for identifying patients with a neutrophil count <1,500/μL at the end of the first treatment cycle ········ 17

Supplementary Table S10. Participant clinical characteristics stratified by the serum BUN level ········ 18

Supplementary Figure S1. PBPK model-predicted and observed plasma concentration–time profiles of pemetrexed in each GFR group ········ 19

Supplementary Figure S2. Correlations of PBPK-derived AUC_{0-24} and AUC_{0-inf} with pemetrexed dose and eGFR, and between the two exposure metrics ········ 20

Supplementary Figure S3. Goodness-of-fit plots for the final model and models with platinum type forced into different model parameters ········ 21

Supplementary Figure S4. Individual fits of the final model in the development cohort ········ 23

Supplementary Figure S5. Correlations between pemetrexed dose and patient characteristics ········ 24

Supplementary Text S1. NONMEM control stream for the final model ········ 25

Supplementary Table S1. Physicochemical and biochemical parameters used in the PBPK model

Property	Value	Reference
Compound name	Pemetrexed	
Molecular weight	427.4	https://pubchem.ncbi.nlm.nih.gov/compound/Pemetrexed[1]
Fat-solubility (LogP)	-0.797	Posada M, et al. 2015[24]
pKa (acid)	3.23, 4.40	Posada M, et al. 2015[24]
Fraction unbound plasma (*f*u)	0.19	Posada M, et al. 2015[24]
Solubility	89.4 mg/mL	https://pubchem.ncbi.nlm.nih.gov/compound/Pemetrexed[1]
GFR fraction	4.50	Fitted in PK-Sim
K_m OAT3	18.2 µM	Posada M, et al. 2015[24]
V_{max} OAT3	185 pmol/min per 10^6 cells	Posada M, et al. 2015[24]

GFR, glomerular filtration rate; OAT3, organic anion transporter 3; PBPK, physiologically based pharmacokinetic.

Supplementary Table S2. Sampling settings for virtual populations employed in the PBPK simulations

	The literature reporting the population referenced in the generation of the virtual populations				
	Nakagawa K, et al. 2006[26]	Kavathiya K, et al. 2017[27]	Mita AC, et al. 2006[28]: GFR ≥ 80 (mL/min)	Mita AC, et al. 2006[28]: GFR 60-79 (mL/min)	Mita AC, et al. 2006[28]: GFR 40-59 (mL/min)
Number of individuals*	31	24	18	13	15
Race*	Japanese	East Asian	White American	White American	White American
Proportion of females (%)*	35	33	11.1	30.8	46.7
Age (year)*	57.00	54.83	56.50	62.00	67.00
Height (cm)**	168.99	166.97	175.55	174.97	174.13
Weight (kg)**	61.87	58.96	83.50	82.89	81.23
BMI (kg/m^2)**	21.67	21.15	27.09	27.07	26.79
GFR (ml/min)	114.2**	99.80**	112.0*	67.20*	50.80*

BMI, body mass index; GFR, glomerular filtration rate.

*Set to match the mean values or applicable categories of the study populations in each literature.

**Set to the values provided by PK-Sim depending on the race, proportion of females, and mean age of each study population.

Supplementary Table S3. Evaluation of the predictive performance of the PBPK model without consideration for GFR levels

Clinical Trial	Dose (mg/m^2)	Route of Administration		CL (mL/min)	C_{max} (μg/mL)	$AUC_{0–inf}$ (μg·h/mL)
Nakagawa K, et al. 2006[26]	300	i.v. infusion (10 min)	Predicted value	85.4	64.9	106
			Observed value[27]	109	58.2	70.1
			AFE	0.78	1.11	1.51
Nakagawa K, et al. 2006[26]	500	i.v. infusion (10 min)	Predicted value	84.8	108	178
			Observed value[27]	86.5	115	158
			AFE	0.98	0.94	1.13
Kavathiya K, et al. 2017[27]	500	i.v. infusion (10 min)	Predicted value	68.4	111	197.88
			Observed value[28]	78.7	113	223.64
			AFE	0.87	0.98	0.88
Nakagawa K, et al. 2006[26]	600	i.v. infusion (10 min)	Predicted value	84.1	130	214
			Observed value[27]	53.0	178	290
			AFE	1.59	0.73	0.74
Nakagawa K, et al. 2006[26]	700	i.v. infusion (10 min)	Predicted value	84.1	152	250
			Observed value[27]	83.4	172	250
			AFE	1.01	0.88	1.00
Nakagawa K, et al. 2006[26]	800	i.v. infusion (10 min)	Predicted value	84.1	174	287
			Observed value[27]	61.4	240	361
			AFE	1.37	0.72	0.79

Nakagawa K, et al. 2006[26]	900	i.v. infusion (10 min)	Predicted value	84.1	196	323
			Observed value[27]	68.5	217	388
			AFE	1.23	0.90	0.83
Nakagawa K, et al. 2006[26]	1000	i.v. infusion (10 min)	Predicted value	83.5	217	360
			Observed value[27]	79.3	269	382
			AFE	1.05	0.81	0.94
Nakagawa K, et al. 2006[26]	1200	i.v. infusion (10 min)	Predicted value	83.5	261	433
			Observed value[27]	99.7	212	337
			AFE	0.84	1.23	1.28
			Geometric mean of AFE (95% CI)	1.05 (0.88–1.26)	0.91 (0.79–1.04)	0.99 (0.82–1.18)

AFE, average fold error; $AUC_{0\text{–}inf}$, area under the plasma concentration–time curve from time zero to infinity; CI, confidence interval; CL, clearance; C_{max}, maximum plasma concentration; GFR, glomerular filtration rate; i.v., intravenous; PBPK, physiologically based pharmacokinetic.

Supplementary Table S4. Evaluation of the predictive performance of the PBPK model with consideration for GFR levels

Clinical Trial	GFR (mL/min)	Dose (mg/m^2)	Route of Administration		CL (mL/min)	C_{max} (µg/mL)	AUC_{0-inf} (µg·h/mL)
Mita AC, et al. 2006[28]	≥ 80	500	i.v. infusion (10 min)	Predicted value	96.9	100	191
				Observed value[29]	93.2	131	188
				AFE	1.04	0.76	1.02
Mita AC, et al. 2006[28]	≥ 80	600	i.v. infusion (10 min)	Predicted value	96.9	120	230
				Observed value[29]	95.0	153	228
				AFE	1.02	0.78	1.01
Mita AC, et al. 2006[28]	60–79	500	i.v. infusion (10 min)	Predicted value	64.7	107	295
				Observed value[29]	69.6	136	228
				AFE	0.93	0.79	1.29
Mita AC, et al. 2006[28]	60–79	600	i.v. infusion (10 min)	Predicted value	63.8	128	355
				Observed value[29]	58.1	138	373
				AFE	1.10	0.93	0.95
Mita AC, et al. 2006[28]	40–59	400	i.v. infusion (10 min)	Predicted value	43.9	83.1	307
				Observed value[29]	54.4	94.9	235
				AFE	0.81	0.88	1.31
Mita AC, et al. 2006[28]	40–59	500	i.v. infusion (10 min)	Predicted value	43.9	104	386
				Observed value[29]	54.7	91.4	300
				AFE	0.80	1.14	1.29

	Geometric mean of AFE (95% CI)	0.94 (0.82–1.09)	0.87 (0.74–1.02)	1.13 (0.97–1.33)

AFE, average fold error; $AUC_{0–inf}$, area under the plasma concentration–time curve from time zero to infinity; CL, clearance; C_{max}, maximum plasma concentration; GFR, glomerular filtration rate; i.v., intravenous; PBPK, physiologically based pharmacokinetic.

Supplementary Table S5. Effects of the tested covariates on the objective values in univariate analysis

Tested covariates	ΔOFV	*P* value
A1		
Platinum type		
Pemetrexed + Cisplatin vs. Pemetrexed + Carboplatin	-2.559	NS
Pemetrexed dose (mg)	-31.399	< 0.001
Pemetrexed dose per BSA (mg/m^2)	-32.371	< 0.001
CL (mL/min): continuous variable	-2.993	NS
CL (mL/min): categorical variable		
< 50 vs. 50 ≤	0	NS
< 60 vs. 60 ≤	-0.332	NS
< 70 vs. 70 ≤	-1.232	NS
C_{max} (μg/mL): continuous variable	-0.258	NS
C_{max} (μg/mL): categorical variable		
< 100 vs 100 ≤	-0.427	NS
< 110 vs 110 ≤	-0.122	NS
$AUC_{0–inf}$ (μg·h/mL): continuous variable	-0.152	NS
$AUC_{0–inf}$ (μg·h/mL): categorical variable		
< 175 vs. 175 ≤	-0.263	NS
< 200 vs. 200 ≤	-0.873	NS
< 225 vs. 225 ≤	-0.976	NS
< 250 vs. 250 ≤	-0.018	NS
$AUC_{0–24}$ (μg·h/mL): continuous variable	2.521	–
$AUC_{0–24}$ (μg·h/mL): categorical variable		
< 175 vs. 175 ≤	0.240	–
< 200 vs. 200 ≤	2.092	–
< 225 vs. 225 ≤	-0.007	NS
< 250 vs. 250 ≤	-0.082	NS
Age (year)	-1.058	NS

BMI (kg/m^2)	-2.192	NS
BSA (m^2)	-1.477	NS
CLcr (mL/min): continuous variable	-0.883	NS
CLcr (mL/min): categorical variable		
< 60 vs. 60 ≤	-0.065	NS
< 60 vs. 60-90 vs. 90 ≤	-1.680	NS
eGFR (mL/min): continuous variable	-0.678	NS
eGFR (mL/min): categorical variable		
< 90 vs. 90 ≤	-1.089	NS
< 60 vs. 60-90 vs. 90 ≤	-1.223	NS
BUN (mg/dL)	-4.300	0.038
NSAIDs	-0.360	NS
RAS inhibitor	-0.295	NS
Diuretics	-0.004	NS
A2		
Platinum type		
Pemetrexed + Cisplatin vs. Pemetrexed + Carboplatin	-3.479	NS
Pemetrexed dose (mg)	-0.179	NS
Pemetrexed dose per BSA (mg/m^2)	-0.079	NS
CL (mL/min): continuous variable	-0.964	NS
CL (mL/min): categorical variable		
< 50 vs. 50 ≤	1.897	–
< 60 vs. 60 ≤	2.746	–
< 70 vs. 70 ≤	-0.185	NS
C_{max} (μg/mL): continuous variable	2.108	–
C_{max} (μg/mL): categorical variable		
< 100 vs. 100 ≤	-2.618	NS
< 110 vs. 110 ≤	-0.403	NS
$AUC_{0–inf}$ (μg·h/mL): continuous variable	-1.971	NS
$AUC_{0–inf}$ (μg·h/mL): categorical variable		

< 175 vs. 175 ≤	-1.286	NS
< 200 vs. 200 ≤	-1.904	NS
< 225 vs. 225 ≤	-2.016	NS
< 250 vs. 250 ≤	-0.028	NS
$AUC_{0–24}$ (μg·h/mL): continuous variable	-1.934	NS
$AUC_{0–24}$ (μg·h/mL): categorical variable		
< 175 vs. 175 ≤	-1.263	NS
< 200 vs. 200 ≤	-1.491	NS
< 225 vs. 225 ≤	-0.244	NS
< 250 vs. 250 ≤	-0.523	NS
Age (year)	-3.455	NS
BMI (kg/m^2)	-4.019	0.045
BSA (m^2)	-1.489	NS
CLcr (mL/min): continuous variable	-0.002	NS
CLcr (mL/min): categorical variable		
< 60 vs. 60 ≤	-2.548	NS
< 60 vs. 60-90 vs. 90 ≤	-2.74	NS
eGFR (mL/min): continuous variable	-1.046	NS
eGFR (mL/min): categorical variable		
< 90 vs. 90 ≤	-0.031	NS
< 60 vs. 60-90 vs. 90 ≤	-0.070	NS
BUN (mg/dL)	-2.881	NS
NSAIDs	-0.072	NS
RAS inhibitor	-5.147	0.023
Diuretics	-2.383	NS
B		
Platinum type		
Pemetrexed + Cisplatin vs. Pemetrexed + Carboplatin	-0.531	NS
Pemetrexed dose (mg)	-14.403	< 0.001
Pemetrexed dose per BSA (mg/m^2)	-16.665	< 0.001

CL (mL/min): continuous variable	-0.004	NS
CL (mL/min): categorical variable		
< 50 vs. 50 ≤	-0.113	NS
< 60 vs. 60 ≤	-0.036	NS
< 70 vs. 70 ≤	-0.123	NS
C_{max} (μg/mL): continuous variable	-1.585	NS
C_{max} (μg/mL): categorical variable		
< 100 vs 100 ≤	-1.938	NS
< 110 vs 110 ≤	-0.297	NS
$AUC_{0–inf}$ (μg·h/mL): continuous variable	-1.002	NS
$AUC_{0–inf}$ (μg·h/mL): categorical variable		
< 175 vs. 175 ≤	-1.867	NS
< 200 vs. 200 ≤	-2.205	NS
< 225 vs. 225 ≤	-2.442	NS
< 250 vs. 250 ≤	2.700	–
$AUC_{0–24}$ (μg·h/mL): continuous variable	-1.134	NS
$AUC_{0–24}$ (μg·h/mL): categorical variable		
< 175 vs. 175 ≤	-4.406	0.036
< 200 vs. 200 ≤	-2.283	NS
< 225 vs. 225 ≤	-0.168	NS
< 250 vs. 250 ≤	-0.596	NS
Age (year)	-1.656	NS
BMI (kg/m^2)	-2.335	NS
BSA (m^2)	-0.069	NS
CLcr (mL/min): continuous variable	-0.022	NS
CLcr (mL/min): categorical variable		
< 60 vs. 60 ≤	-0.055	NS
< 60 vs. 60-90 vs. 90 ≤	-0.090	NS
eGFR (mL/min): continuous variable	-0.021	NS
eGFR (mL/min): categorical variable		

< 90 vs. 90 ≤	-0.470	NS
< 60 vs. 60-90 vs. 90 ≤	-0.09	NS
BUN (mg/dL)	-2.433	NS
NSAIDs	-3.168	NS
RAS inhibitor	-0.054	NS
Diuretics	-0.054	NS

$AUC_{0–inf}$, area under the plasma concentration–time curve from time zero to infinity; $AUC_{0–24}$, area under the plasma concentration–time curve from time zero to 24 h; BMI, body mass index; BSA, body surface area; BUN, blood urea nitrogen; CL, clearance; CLcr, creatinine clearance; C_{max}, maximum plasma concentration; eGFR, estimated glomerular filtration rate; NS, no significant; NSAIDs, non-steroidal anti-inflammatory drugs; OFV, objective function value; RAS, renin–angiotensin system.

Supplementary Table S6. Summary of changes in objective values in stepwise covariate modeling

Model description	OFV	ΔOFV	*P* value
Base model	630.565	–	–
Forward step			
Add RAS inhibitors on A2	625.418	-5.147	0.023
Add $AUC_{0–24}$ (< 175 vs. 175 ≤) on B	618.603	-6.815	0.009
Add BUN on A1	613.932	-4.671	0.031
Add BMI on A2	614.193	0.261	–
Full model	613.932	–	–
Backward step			
Eliminate the RAS inhibitor on A2	620.297	6.365	0.012
Eliminate $AUC_{0–24}$ (< 175 vs. 175 ≤) on B	620.723	6.791	0.009
Eliminate BUN on A1	618.603	4.671	0.031

$AUC_{0–24}$, area under the plasma concentration–time curve from time zero to 24 h; BMI, body mass index; BUN, blood urea nitrogen; OFV, objective function value; RAS, renin–angiotensin system.

Supplementary Table S7. Parameter estimates of the NLME models and bootstrap analysis

	Final model			Bootstrap analysis	
Parameter	Estimate	RSE (%)	Shrinkage (%)	Median	95% CI
The model obtained in Step 1					
Estimated population mean					
A1	0.717	24.7	–	0.744	0.408–1.19
A2	0.0319	43.3	–	0.0286	0.00280–0.0962
B	1.32	11.7	–	1.32	1.05–1.74
Effect of covariate on A1					
Pemetrexed dose	1.27	24.4	–	0.853	0.0552–2.90
Interindividual variability					
ω_{A1}	0.763	28.5	18.7	0.752	0.468–0.971
ω_{A2}	1.59	47.0	36.3	1.74	1.17–3.72
ω_{B}	0.187	105	58.4	0.206	0.0815–0.364
Residual variability					
ε	0.262	18.4	14.6	0.259	0.212–0.291
The model obtained in Step 2					
Estimated population mean					
A1	0.334	36.2	–	0.365	0.127–0.688
A2	0.0443	63.0	–	0.0369	0.00302–0.863
B	1.39	18.5	–	1.40	0.848–3.05
Effect of covariate on A1					
BUN	-0.975	40.5	–	-0.882	-1.69 to -0.185
Effect of covariate on A2					
RAS inhibitor	0.0451	57.2	–	0.0699	0.0164–0.481
Effect of covariate on B					
$AUC_{0–24}$	1.44	19.0	–	1.36	1.01–2.31
Interindividual variability					
ω_{A1}	0.835	23.5	15.9	0.786	0.443–1.08
ω_{A2}	1.96	46.6	49.8	2.11	0.814–4.56
ω_{B}	0.148	103	67.9	0.165	0.00235–0.315
Residual variability					
ε	0.271	12.2	11.0	0.269	0.238–0.297

$AUC_{0–24}$, area under the plasma concentration–time curve from time zero to 24 h; BUN, blood urea nitrogen; CI, confidence interval; NLME, nonlinear mixed-effects; RAS, renin–angiotensin system; RSE, relative standard error.

Supplementary Table S8. Changes in the objective function value after adding platinum type as a covariate to different model parameters

Model description	OFV	ΔOFV	*P* value
Final model	613.932	–	–
Add Platinum type on A1 (forced entry)	613.254	-0.678	0.410
Add Platinum type on A2 (forced entry)	618.384	4.452	–
Add Platinum type on B (forced entry)	617.088	3.156	–

OFV, objective function value.

Supplementary Table S9. Discriminative performance of the final model for identifying patients with a neutrophil count <1,500/μL at the end of the first treatment cycle

Cohort	AUROC (95% CI)	Sensitivity (95% CI)	Specificity (95% CI)	PPV (95% CI)	NPV (95% CI)
Development cohort	0.768 (0.639–0.898)	0.900 (0.600–1.000)	0.671 (0.382–0.882)	0.270 (0.175–0.474)	0.981 (0.942–1.000)
Validation cohort	0.718 (0.545–0.891)	1.000 (0.800–1.000)	0.564 (0.461–0.910)	0.128 (0.106–0.364)	1.000 (0.985–1.000)

AUROC, area under the receiver operating characteristic curve; CI, confidence interval; NPV, negative predictive value; PPV, positive predictive value.

Supplementary Table S10. Participant clinical characteristics stratified by the serum BUN level

	Low BUN (n = 43)	High BUN (n = 43)	*P* value
Age (year)	65.47 ± 7.59	71.02 ± 7.15	< 0.001
Sex (male/female)	29 (67.4) /14 (32.6)	33 (76.7) /10 (23.3)	0.471
Height (cm)	160.83 ± 9.87	162.71 ± 8.27	0.340
Weight (kg)	57.48 ± 11.85	59.81 ± 10.10	0.331
BMI (kg/ m^2)	22.10 ± 3.61	22.58 ± 3.41	0.532
BSA (m^2)	1.59 ± 0.19	1.64 ± 0.16	0.275
Creatinine (mg/dL)	0.71 ± 0.13	0.81 ± 0.18	0.005
CLcr (mL/min)	83.16 ± 27.59	71.48 ± 19.29	0.025
eGFR (mL/min)	75.02 ± 21.11	67.16 ± 16.24	0.056
BUN (mg/dL)	11.10 ± 2.30	19.39 ± 3.64	< 0.001
Pemetrexed dose (mg)	755.81 ± 125.27	765.47 ± 130.11	0.727
Pemetrexed dose per BSA (mg/m^2)	463.28 ± 85.79	466.36 ± 55.49	0.844
Co-administration			
Cisplatin/Carboplatin	25 (58.1) / 18 (41.9)	30 (30.2) / 13 (69.8)	0.016
NSAIDs	9 (20.9)	14 (32.6)	0.330
RAS inhibitor	15 (34.9)	10 (23.3)	0.342
Diuretics	6 (14.0)	2 (4.7)	0.265

The data are presented as means ± standard deviations or proportions for categorical variables.

BUN was dichotomized by the median of 15 mg/dL.

BMI, body mass index; BSA, body surface area; BUN, blood urea nitrogen; CLcr, creatinine clearance; NSAIDs, non-steroidal anti-inflammatory drugs; RAS, renin–angiotensin system.

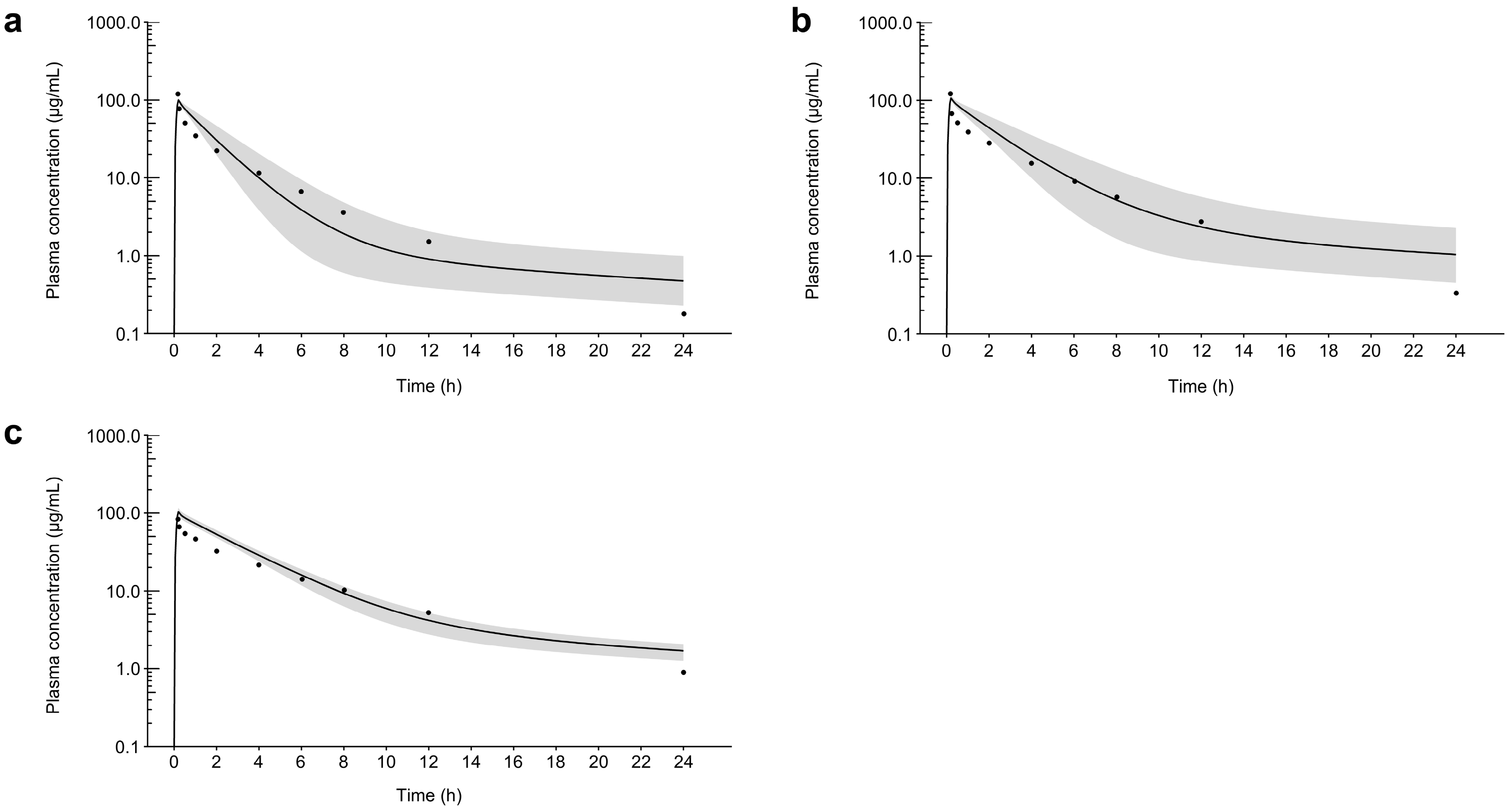

Supplementary Figure S1. PBPK model-predicted and observed plasma concentration–time profiles of pemetrexed in each GFR group

The predicted and observed plasma concentration–time profiles of pemetrexed for (a) GFR ≥ 80 mL/min, (b) GFR 60–79 mL/min, and (c) GFR 40–59 mL/min. The solid line and shaded area represent the arithmetic mean and 5th–95th percentile range of the virtual population, respectively. Solid circles plot the observed plasma concentrations. References for the reported observations are given in Table S3.

GFR, glomerular filtration rate; and PBPK, physiologically based pharmacokinetic.

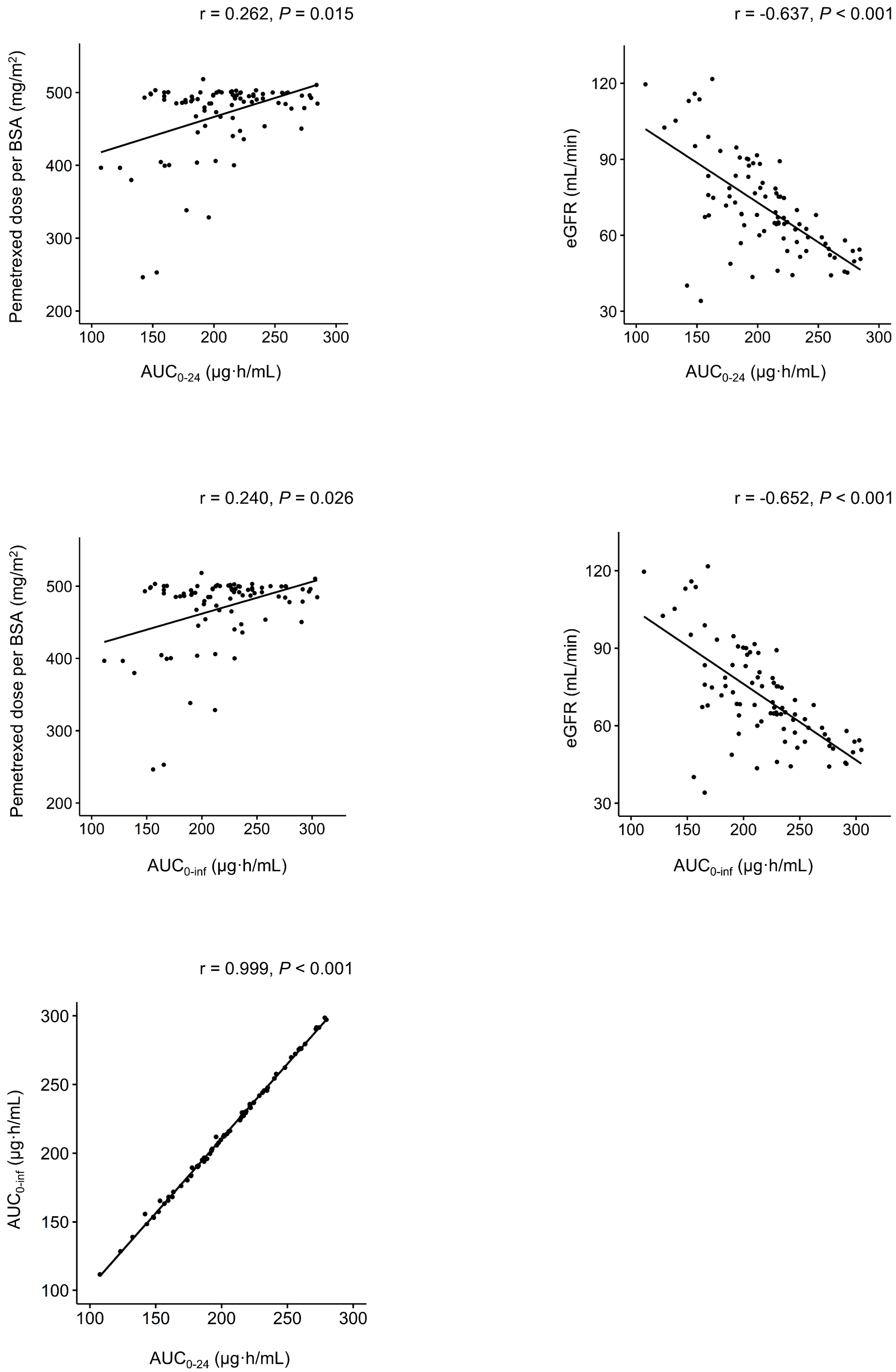

Supplementary Figure S2. Correlations of PBPK-derived $AUC_{0\text{-}24}$ and $AUC_{0\text{-inf}}$ with pemetrexed dose and eGFR, and between the two exposure metrics

$AUC_{0\text{–inf}}$, area under the plasma concentration–time curve from time zero to infinity; $AUC_{0\text{–}24}$, area under the plasma concentration–time curve from time zero to 24 h; eGFR, estimated glomerular filtration rate.

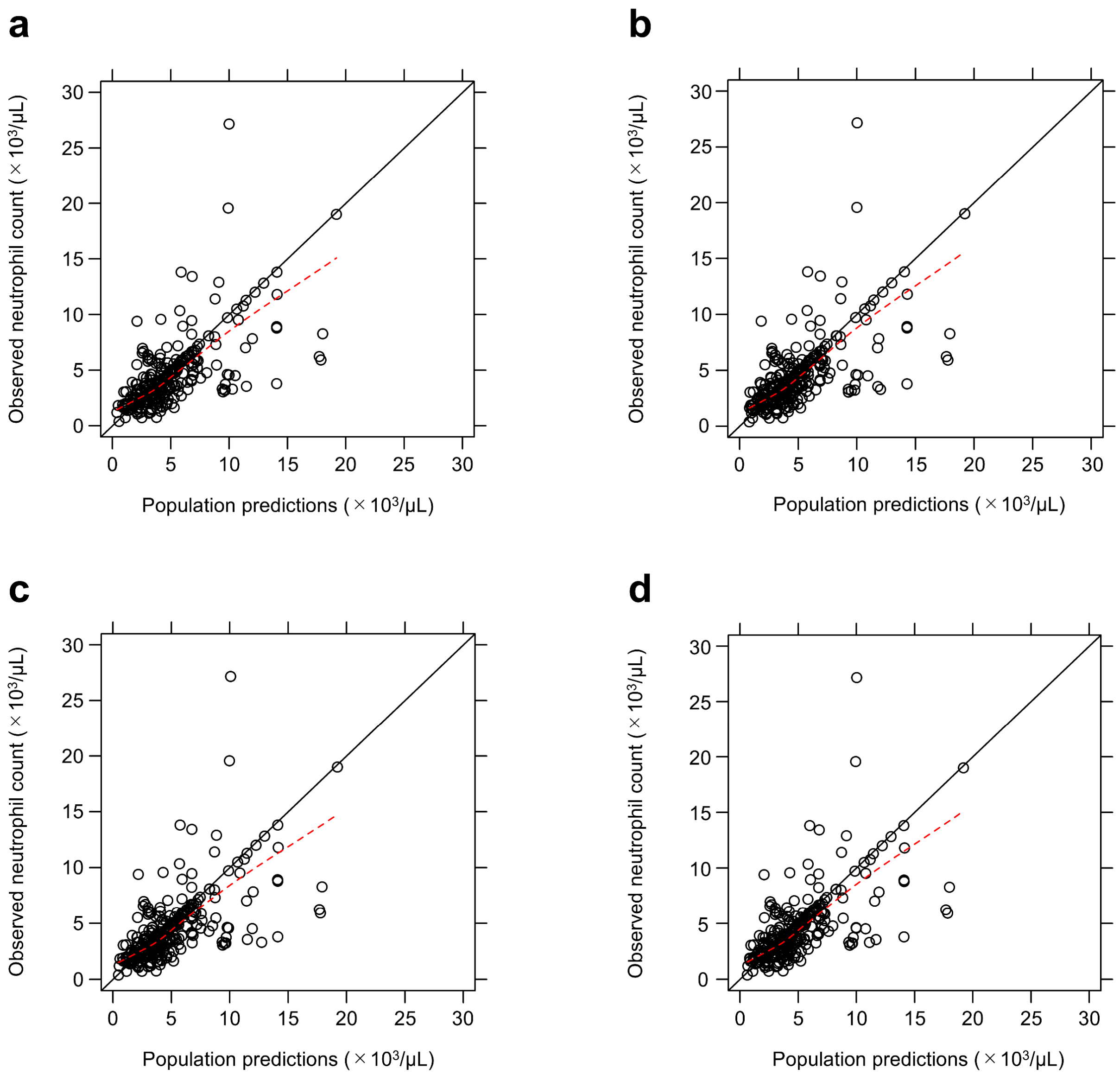

Supplementary Figure S3. Goodness-of-fit plots for the final model and models with platinum type forced into different model parameters

The observed vs. population-predicted neutrophil count for (a) the final model, (b) model with forced inclusion of platinum type on A1, (c) model with forced inclusion of platinum type on A2, and (d) model with forced inclusion of platinum type on B.

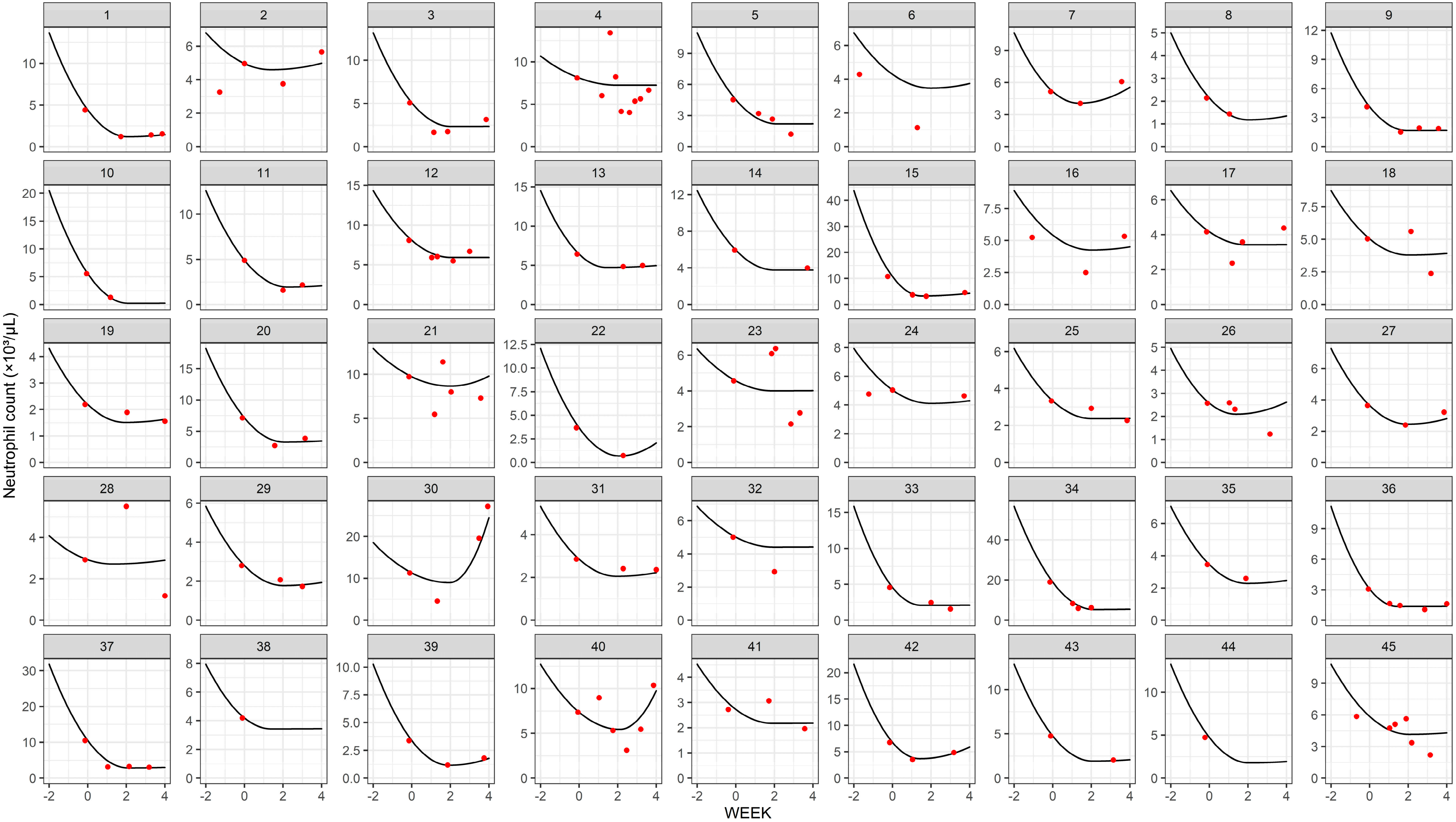

Neutrophil count (×10³/μL)
WEEK
1
2
3
4
5
6
7
8
9
10
11
12
13
14
15
16
17
18
19
20
21
22
23
24
25
26
27
28
29
30
31
32
33
34
35
36
37
38
39
40
41
42
43
44
45

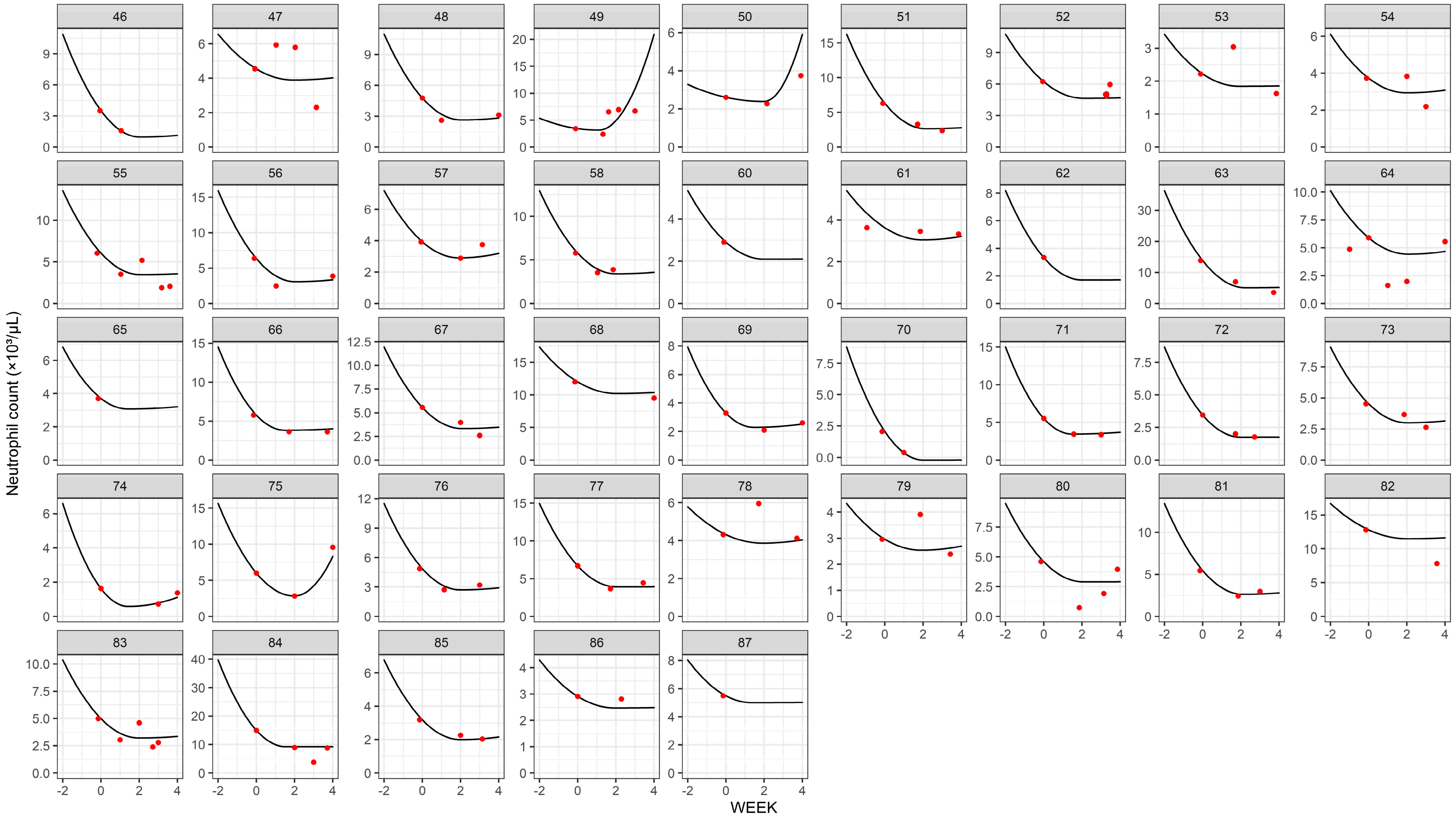

Supplementary Figure S4. Individual fits of the final model in the development cohort

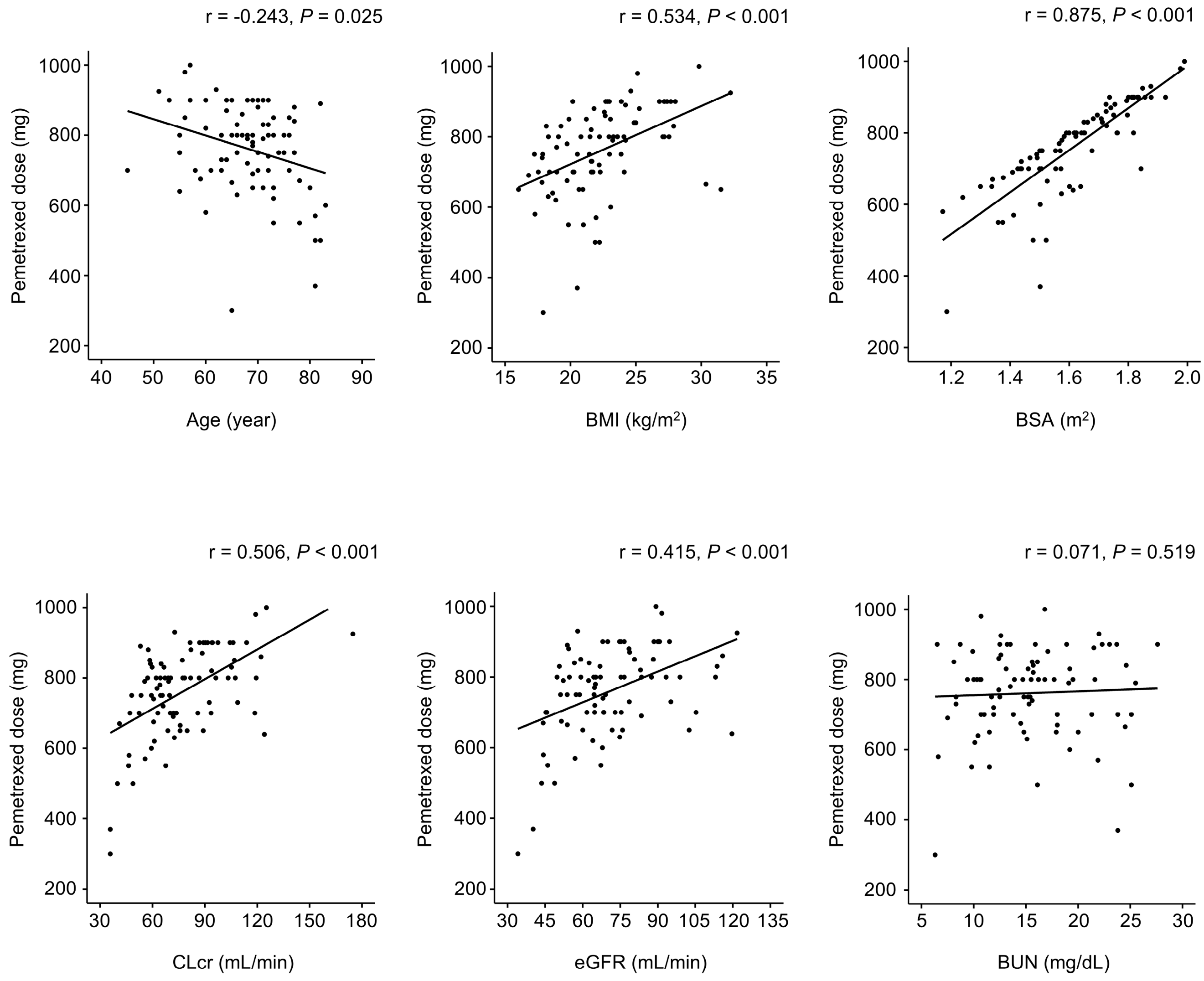

Supplementary Figure S5. Correlations between pemetrexed dose and patient characteristics

Correlation coefficients (r) and *P* values were calculated using Spearman's rank correlation coefficient test.

BMI, body mass index; BSA, body surface area; BUN, blood urea nitrogen; CLcr, creatinine clearance; and eGFR, estimated glomerular filtration rate.

Supplementary Text S1. NONMEM control stream for the final model

```
$PROBLEM PEM

$INPUT
ID EVID DV MDV WEEK BASE SEX AGE Pem_DOSE DOSE_BSA CDDP_DOSE CBDCA_DOSE
AUCinf AUC24 AUC_2 Cmax CL Platinum NSAIDs RAS diuretic SCr eGFR eGFR_2 eGFR_3 CCr
CCr_2 CCr_3 BUN BSA BMI

$DATA PEM2024.csv
IGNORE=#

$PRED
  BUNP = (BUN/15.2) ** THETA(4)

  ras1=0
  ras2=0
   IF(RAS.EQ.0) ras1=1
   IF(RAS.EQ.1) ras2=1
      rasP = ras1 + ras2 * THETA(5)

  AUC1=0
  AUC2=0
   IF(AUC_2.EQ.0) AUC1=1
   IF(AUC_2.EQ.1) AUC2=1
      AUCP = AUC1 + AUC2 * THETA(6)

  A1    =   THETA(1) * BUNP * exp( ETA(1))
  A2    =   THETA(2) * rasP * exp( ETA(2))
   B    =   THETA(3) * AUCP * exp( ETA(3))
   C    =   BASE

  N1    =   A1 * ( WEEK - B )**2 + ( C - A1*B**2 )
  N2    =   A2 * ( WEEK - B )**2 + ( C - A1*B**2 )

  if ( WEEK.lt.B) then
   N2 = 0
  else
   N1 = 0
  end if
```

```
    Y        = ( N1 + N2 ) * exp(EPS(1))

    IPRED    =    N1 + N2
    IRED = IPRED - DV

$THETA
  (0, 0.5) ;A1
  (0, 0.0011) ;A2
  (0, 1.5) ;B
  (0, 0.002) ;BUN_A1
  (0, 0.4) ;RAS_A2
  0.006 ;AUC_175_B

$OMEGA
  0.2    ;A1
  0.01    ;A2
  0.01    ;B

$SIGMA
  0.01    ;Y

$ESTIMATION NOABORT MAXEVAL=9999 PRINT=0 METHOD=COND INTER
$COVARIANCE PRINT=E
$TABLE            NOPRINT ONEHEADER FILE=SIM.csv FORMAT=,F10.3 ID WEEK DV
                BASE A1 A2 B Pem_DOSE Platinum Y IPRED IRED
```
